# Functional diversification of yeast telomere associated protein, Rif1, in higher eukaryotes

**DOI:** 10.1186/1471-2164-13-255

**Published:** 2012-06-19

**Authors:** Easwaran Sreesankar, Ramamoorthy Senthilkumar, Vellaichamy Bharathi, Rakesh K Mishra, Krishnaveni Mishra

**Affiliations:** 1 Department of Biochemistry, School of Life Sciences, University of Hyderabad, Hyderabad, 500046, India; 2 Centre for Cellular and Molecular Biology, Council of Scientific and Industrial Research, Uppal Road, Hyderabad, 500 007, India

## Abstract

**Background:**

Telomeres are nucleoprotein complexes at the end of linear eukaryotic chromosomes which maintain the genome integrity by regulating telomere length, preventing recombination and end to end fusion events. Multiple proteins associate with telomeres and function in concert to carry out these functions. Rap1 interacting factor 1 (Rif1), was identified as a protein involved in telomere length regulation in yeast. Rif1 is conserved upto mammals but its function has diversified from telomere length regulation to maintenance of genome integrity.

**Results:**

We have carried out detailed bioinformatic analyses and identified Rif1 homologues in 92 organisms from yeast to human. We identified Rif1 homologues in *Drosophila melanogaster*, even though fly telomeres are maintained by a telomerase independent pathway. Our analysis shows that *Drosophila* Rif1 (dRif1) sequence is phylogenetically closer to the one of vertebrates than yeast and has identified a few Rif1 specific motifs conserved through evolution. This includes a Rif1 family specific conserved region within the HEAT repeat domain and a motif involved in protein phosphatase1 docking. We show that dRif1 is nuclear localized with a prominent heterochromatin association and unlike human Rif1, it does not respond to DNA damage by localizing to damaged sites. To test the evolutionary conservation of dRif1 function, we expressed the dRif1 protein in yeast and HeLa cells. In yeast, dRif1 did not perturb yeast Rif1 (yRif1) functions; and in HeLa cells it did not colocalize with DNA damage foci.

**Conclusions:**

Telomeres are maintained by retrotransposons in all *Drosophila* species and consequently, telomerase and many of the telomere associated protein homologues are absent, including Rap1, which is the binding partner of Rif1. We found that a homologue of yRif1 protein is present in fly and dRif1 has evolutionarily conserved motifs. Functional studies show that dRif1 responds differently to DNA damage, implying that dRif1 may have a different function and this may be conserved in other organisms as well.

## Background

Telomeres are nucleoprotein structures found at the ends of linear chromosomes and are critical for genome stability. In most eukaryotes, telomeric DNA consists of multiple copies of simple sequences ranging from a few hundred to a few thousand base pairs. These sequences are usually G rich at the 3′ end and are extended by a specialized, self-templated reverse transcriptase, the telomerase. Telomeres play two important roles: (1) they serve as substrates for telomerase and thus prevent the loss of sequences at the very end as would be expected for a linear sequence replicated by semi-conservative DNA replication. This process is also precisely controlled in such a manner that only a designated amount of repeats are added and no uncontrolled elongation takes place. (2) They protect the ends from being recognized as double-strand breaks and from being attacked by nucleases. All these functions are carried out by multiple proteins that associate with the telomeres (reviewed in [1-4]).

Rif1 (Rap1 interacting factor) was identified in yeast *Saccharomyces cerevisiae*, as an interactor of the major telomere repeat sequence binding protein Rap1 [5]. Rif1 is a negative regulator of telomerase and together with another Rap1 interacting protein, Rif2, it controls the access of telomerase to telomere ends for replication and elongation of telomere sequences [6,7]. Accordingly, *rif1* mutants have abnormally elongated telomeres. Furthermore, in the absence of telomerase, Rif1 inhibits the production of “Type II” survivors, which use the Rad50 dependent recombination pathway to generate telomeres [8]. In yeast, Rif1 protein has been localized predominantly to telomeres where it also antagonizes the establishment of silent chromatin [9-11].

Given the key role of Rif1 in telomere biology, Rif1 homologues have been identified in other yeasts as well. In *Schizosaccharomyces pombe*, the Rif1 orthologue, is recruited to telomeres via another telomere sequence binding protein Taz1 and *rif1* mutants have moderately elongated telomeres, suggesting that it is a negative regulator of telomere length [12]. However, as *rif1* mutants in *S. pombe* show additive telomere length defects in *rap1* mutants, it may work with Taz1 in a parallel pathway with Rap1 to control telomere length [13]. Furthermore, Rif1 has no effect on telomeric heterochromatin establishment in *S. pombe*. Recently, Rif1 orthologue from another budding yeast, *Candida glabrata*, has been studied. Although the effect on telomere length control by Rif1 was not reported, it was shown that in *C. glabrata*, Rif1 is essential for subtelomeric silencing [14]

Presence of Rif1 orthologues in vertebrates points to the key role of this protein in eukaryotes. Rif1 was first identified in mouse and was shown to be expressed at very high levels in totipotent and pluripotent cells, testes and was also associated with telomeres [15]. Subsequently, human Rif1 (hRif1) was identified and these studies suggested a divergence in the functions of Rif1 [16,17]. hRif1 associated with damaged DNA, including dysfunctional telomeres. Further studies established that hRif1 colocalized with several other DNA-damage response factors and depletion of hRif1 led to radiation sensitivity and defects in S-phase checkpoint. Additionally, through depletion studies in mouse cells, it has been demonstrated that mRif1 is essential and that it is involved in repair of stalled replication forks by homology directed repair [18]. hRif1 is upregulated in breast tumours and is proposed to be an anti-apoptotic factor required for DNA repair [19]. More recently, hRif1 was copurified with BLM helicase and was proposed to provide a DNA binding interface for recruiting factors involved in initiation of replication at stalled forks [20].

The studies from yeast to mammals show that Rif1 function has evolved from a protein that specifically participated in replication of the special DNA sequences present at the telomeres to a more general role in DNA damage response and reinitiation of replication at stalled replication forks. *Drosophila*, unlike mammals and yeasts, does not have simple sequence repeats at the telomeres. Instead they maintain their telomeres through the transposition of specialized non-LTR retroposons, namely, HeT-A, TART and TAHRE [21]. A putative Rif1 homologue in *Drosophila* has been reported based on sequence similarities to yeast Rif1 though its function has not been tested [12,16,20]. The presence of a Rif1 homologue in *Drosophila* suggests an early evolution of this telomeric protein to perform non-telomere related functions.

We performed a detailed bioinformatic analysis of *Drosophila* Rif1 (dRif1) to understand the evolutionary history of this protein. We found that Rif1 is conserved in all eukaryotes and dRif1 is closer to vertebrate Rif1 than yeast. A few conserved motifs were identified in the protein which will be helpful in elucidating the molecular basis of its function. We have followed the bioinformatic analyses with experimental test of conserved functions. We find that *Drosophila* and vertebrate Rif1 differ in their interaction with yeast telomeres and their response to DNA damage. Our data suggest that this protein has acquired additional domains in vertebrates and consequently additional roles.

## Results

### Rif1 homologues are conserved across eukaryotes

**Figure 1:**
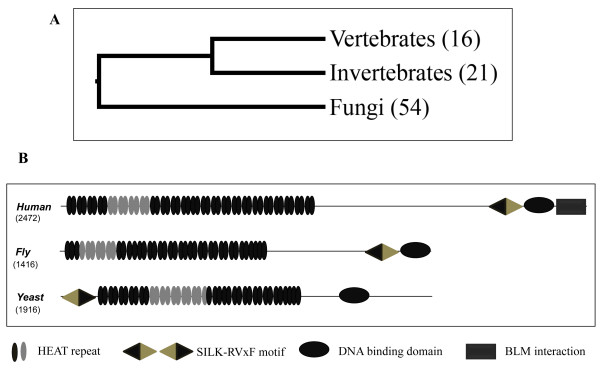
** A) The phylogenetic tree of Rif1 homologues.** The simplified version of the phylogenetic tree of Rif1 homologues (The detailed tree is shown in the Additional file 2). A common branching is seen in three major classes (Fungi, Invertebrates and Vertebrates) and the number of organisms from each branch having the Rif1 homologues is mentioned in the parentheses. **B) The conserved domains of Rif1 homologues.** The conserved domains of Rif1 homologues of human, fly and yeast are shown. The protein length is mentioned below the organism name. The conserved domains are highlighted in different shapes (SILK/PP1 interaction domain – diamond, DNA binding domain –oval (Horizontal), BLM interaction domain –rectangle, HEAT repeat – oval (vertical) and the core conserved region of HEAT repeat is highlighted in grey). The motifs are mapped approximately to the scale.

The Rif1 protein sequence of human and yeast were used for finding the homologues in NCBI protein sequence database. By this approach we found Rif1 homologues in 92 different organisms, including 54 fungal species, 18 insects and 16 vertebrate species (Additional file 1). In addition, we found the homologues in *Hydra magnipapillata* (Cnidarian), *Trichoplax adhaerens* (Placozoan) and *Saccoglossus kowalevskii* (Hemichordata). Phylogenetic tree constructed using the protein sequences of Rif1 shows an evolutionary pattern from lower to higher organisms (Figure 1A and Additional file 2) and indicates that the insect homologues are closer to human than fungal Rif1. We did not find clear homologues of Rif1 in plants, although a related protein in a lycophyte, *Selaginella moellendorffii*, was detected. While search with this lycophyte protein sequence in plants returned several uncharacterized proteins showing reasonable similarity (Additional file 3), these proteins lack the key conserved SILK/PP1 interaction domain (see below). We therefore deemed the plant homologues to be too diverged for further analysis.

### Conserved motifs in Rif1 homologues

We found three motifs, namely, HEAT repeat, SILK motif and a domain present in the C-terminal end which was shown to have DNA binding property [20], that are conserved across the species from yeast to mammals in Rif1 (Figure 1B). In addition, previously predicted BLM helicase interaction domain is conserved only in the vertebrates [20]. HEAT repeat is a structural domain with poor sequence homology and is present in several proteins [22]. It spans ~1000 amino acids in Rif1 homologues [20]. In our detailed analysis we found a highly conserved region of 101–149 amino acids present within the HEAT repeat that is Rif1 specific (Additional file 4). This domain is also present in the putative homologues identified in plants.

**Figure 2:**
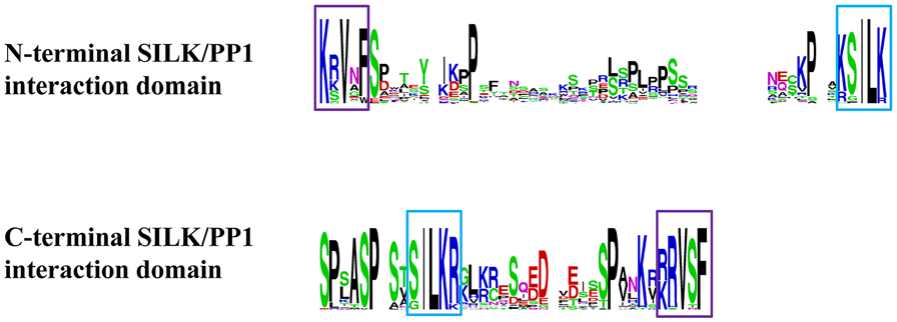
** The consensus pattern of SILK/PP1 interaction domain.** The consensus pattern of N-terminal and C-terminal SILK/PP1 interaction domains is shown in the figure. The core conserved motifs SILK and RVxF are highlighted in blue and violet coloured boxes. The height of each residue corresponds to the degree of conservation across the homologues.

Our analysis identified another novel feature, SILK motif or Protein Phosphatase (PP1) interaction domain***,*** in all Rif1 homologues (Figure 1B, Additional files 5 and 6). The highly conserved residues RVxF were also detected along with the SILK motif, which is the docking motif essential for PP1 interaction [23,24]. Earlier studies have shown that the SILK motif is specifically associated with RVxF motif in certain class of PP1 interacting proteins [23,25]. In all Rif1 homologues, we found SILK and RVxF combination to be present with varying length of amino acid sequences between them. Recently, a large scale proteomics study revealed that the mammalian Rif1 interacts with PP1 by affinity chromatography [26], indicating that Rif1 is a target of PP1. Interestingly, the SILK-RVxF domain at the N-terminal end of Rif1 homologues of fungi is present at the C-terminal end of multi-cellular eukaryotes (Figure 2). Thus there has been a swapping of SILK motif in Rif1 from N-terminal end to C-terminal end during the course of evolution. This shift is seen from placozoans onwards, which are the basal group of multi-cellular organisms (Additional file 7). Additionally, in single cell organisms, when the SILK motif is seen in the N-terminus its architecture is ‘SILK-RVxF’; but in multi cellular organisms the motif is shifted to C-terminus and the architecture is reversed to ‘RVxF-SILK’(Figure 2). Based on the architecture and position of the SILK domain, we again find that the *Drosophila* homologue is closer to vertebrates than yeasts (Additional file 7). Further analysis of other proteins carrying SILK/PP1 interaction domain in human, yeast and *Drosophila* showed that the internal swapping of the motifs giving the two architectures of this domain is not unique to Rif1 (Additional files 8,9 and 10).

A unique DNA binding domain was reported in hRif1 which helps in bringing the BLM helicase to the stalled replication forks [20]. We found that this domain is conserved from yeast to human (Additional file 11). Although the sequence homology of Rif1 is poor between unicellular and multicellular organisms, the profile based search strongly supports the conservation of this DNA binding domain between these two groups of organisms. BLM interaction domain was also reported in the study of hRif1 by Xu et al. [20]. Our analysis shows that this domain is conserved only in vertebrates (Additional file 12).

In summary, our bioinformatic analyses identified several interesting features of Rif1. We report for the first time the conservation of SILK-RVxF motif in Rif1 from all organisms. We also identify a Rif1 specific core HEAT repeat present in all organisms. The conservation of features of the putative DNA binding domain across species again emphasizes the evolution of the protein from the core sequence and it is important to test if the DNA binding function is also retained.

### dRif1 is localized to the nucleus and is prominently associated with heterochromatin

**Figure 3:**
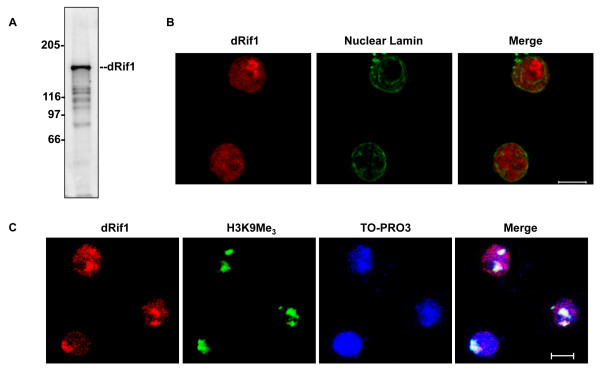
** dRif1 localizes to the nucleus in unperturbed cells. A)** Western blot of total protein extract from S2 cells was probed with antibody against dRif1. A 160 kDa lights up prominently. **B)** S2 cells were immunostained with antibodies to dRif1 (red) and costained with antibodies to lamin (green) to mark the nucleus. **C)** S2 cells were immunostained with antibodies to H3K9 trimethyl (green) and dRif1 (red) and TO-PRO3 to mark the nucleus (scale bar, 5 μm).

In order to functionally characterize dRif1, we raised polyclonal antibodies against a part of the protein. The antibody recognized a protein of approximately 160 kDa, as expected, in *Drosophila* embryo derived S2 cell extract (Figure 3A). We performed immunolocalization of dRif1 in S2 cells to see the subcellular localization, and found that Rif1 was nuclear localized (Figure 3B). dRif1 stained the nucleus in a heterogenous manner, with most nuclei showing one or two prominent dark patches along with a diffuse nuclear staining. As the same regions also appeared to contain dense DNA staining, we tested if this patch corresponded to heterochromatin. We colocalized dRif1 with the heterochromatin marker, histone H3 trimethyl lysine 9. As shown in Figure 3C, we found that dRif1 associates with heterochromatin prominently in S2 cells.

### dRif1 does not relocalize upon DNA damage induction in S2 cells

**Figure 4:**
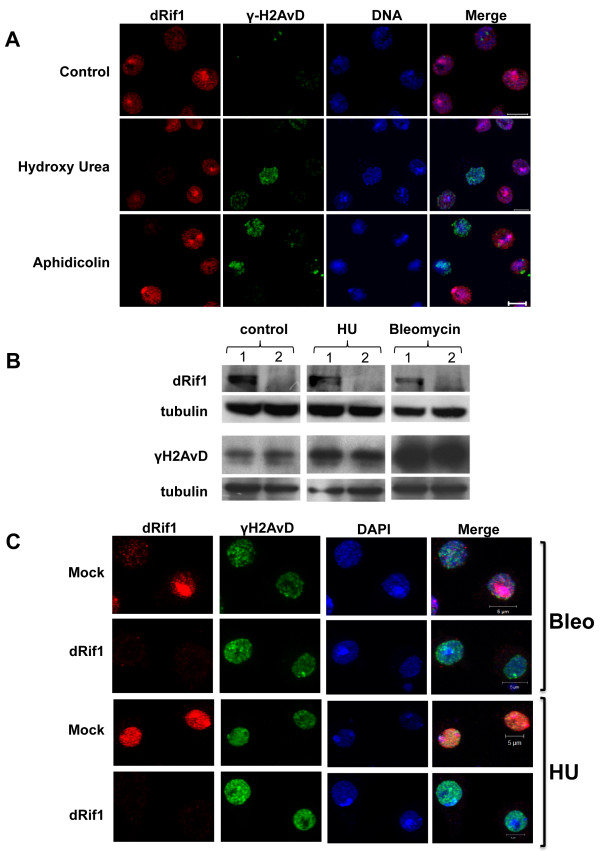
** dRif1 does not colocalize to the DNA damage foci induced by HU and Aphidicolin. A)** S2 cells were treated with either 2.5 mM hydroxy Urea (HU) or 25 μM aphidicolin for 16 hrs to induce DNA damage and then fixed, stained with antibodies to dRif1 (red) and γH2AvD (green). The slides were mounted in mounting media containing DAPI or TO-PRO 3 (blue). **B)** S2 cells were treated with either dsRNA of GFP (1) or dRif1 (2). Each was further split into three parts and was either mock treated or treated with hydroxyurea (HU) or bleomycin. Protein extracts were tested for dRif1 and γH2AvD expression; tubulin was used as a loading control. dRif1 was undetectable in RNAi treated cells. γH2AvD levels increase upon exposure to DNA damage. **C)** The cells were also immunostained with γH2AvD to detect DNA damage sites. All the cells were fixed and stained for dRif1 (red), γH2AvD (green) antibody and DAPI (blue) Scale bar is 5 μm.

Immunolocalization of human Rif1 shows a diffuse nuclear staining. Multiple forms of DNA damage, including ionising radiation, hydroxy urea, MMS, etoposide, aphidicolin cause hRif1 to relocalize into foci, which often coincide with the damage sites [16-19]. To test if dRif1 also responds to damaged DNA in a similar manner, we treated S2 cells with hydroxy urea and aphidicolin and asked if dRif1 relocalized to halted replication forks. Cells were costained with γ-H2AvD antibodies to mark the sites of damaged DNA. In contrast to what has been observed in human cells, we did not see any major relocalization of dRif1 with either hydoxy urea or aphidicolin treatment (Figure 4A). DNA damage foci that showed strong γ-H2AvD staining were prominent in the treated cells showing that treatment did induce DNA damage. Same results were obtained with MMS and UV treatments (data not shown). Therefore, dRif1, unlike hRif1, does not relocalize upon DNA damage.

The experiments described above showed that dRif1 does not respond to DNA damage by localizing to the repair sites. However, in order to test this more directly, we carried out knock down experiments using double-stranded RNA. Three different primer sets with no off targets were designed for dRif1. We used dsRNA of GFP for the mock treatment experiments. We did two successive rounds of dsRNA treatment and performed both western blot and immunofluorescence studies and confirmed that dRif1 protein levels decreased to undetectable levels by the sixth day (Figure 4B). Cells remained healthy and continued to divide for several days after dsRNA treatment. These knockdown cells were treated with DNA damage inducing agents, HU and bleomycin. After treatment we stained the cells for γ-H2AvD and dRif1. First, we did not find any difference in viability between mock treated and double stranded dRif1 RNA treated cells upon induction of DNA damage. Second, upon staining for γ-H2AvD, we found several spots come up on DNA damage induction (Figure 4C). We compared the levels of γ-H2AvD between wild type and knock down cells by western blots (Figure 4B). Our results show, both by immunofluorescence and by western blots, that repair foci (therefore, signaling of DNA damage) occur normally in the absence of dRif1. These data further strengthen our conclusions that unlike human Rif1, *Drosophila* Rif1 does not participate in DNA damage response by translocating to the sites of repair.

### Knock down of dRif1 does not influence telomere transcription in S2 cells

**Figure 5:**
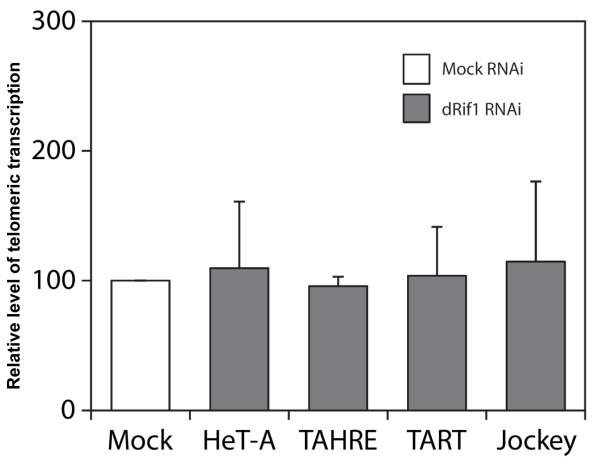
** dRif1 knock down does not affect telomeric transcription in S2 cells.** RNA was extracted from mock and dRif1 dsRNA treated cells and RT-PCR was performed to detect levels of telomere associated retrotransposon transcripts (HeT-A, TAHRE, TART and jockey, the internally located retrotransposon).

As yRif1 represses transcription of telomeric repeat containing transcripts (TERRA) [27,28] and is involved in telomere position effect in *C. glabrata*, we asked if telomere specific transcript levels are under dRif1 control in *Drosophila*. *Drosophila* telomeres have retrotransposon elements that are generally transcriptionally suppressed and are activated by mutations that inactivate telomere position effect and rasiRNA pathway, indicating that they are under strict transcriptional repression (reviewed in [29]). We isolated RNA from wild type and knock down cells and performed quantitative reverse transcriptase PCR for transcripts from telomere associated transposons [30].We do find low levels of transcripts in wild type cells and this level is not significantly affected by knock down of Rif1 (Figure 5). This result suggests that dRif1 is unlikely to regulate telomere retrotransposon transcript levels in S2 cells.

### dRif1 does not complement telomere function in yeast

**Figure 6:**
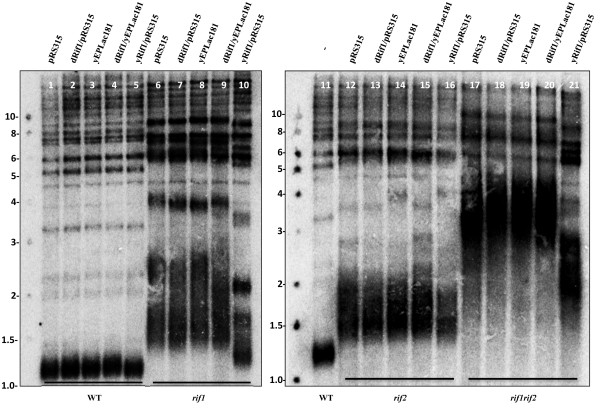
** Expression of dRif1 protein in yeast does not interfere with telomere length maintenance.** Southern blot of telomeric restriction fragments from wild type (KRY-12), *rif1*, *rif2* and *rif1rif2* double mutant yeast strains transformed with either empty vector (pRS315, yEpLac181), yRif1(positive control) or dRif1 in different vectors. XhoI digests of the genomic DNA probed with dGT repeat to identify the telomeric repeat length. The median length of wild type telomeres is approximately 1.2 kb, *rif1* is 2 kb, *rif2* is 1.6 kb and *rif1rif2* is 4 kb.

The bioinformatic analyses presented above show that Rif1 is conserved throughout eukaryotes. However, as shown above, we found that dRif1 responded differently to DNA damage in comparison to human Rif1. To test how much of the core functional properties of yeast Rif1 are retained in *Drosophila,* we performed cross complementation assays. To this end the full length dRif1 was expressed under the control of yeast Rif1 promoter and transformed into wild type, *rif1rif2* and *rif1rif2* mutant yeast strains and the telomere length was estimated. Yeast lacking Rif1p have much longer telomeres than wild type cells [5,6]. As seen in Figure 6, wild type cells have compact telomeres around 1.2 kb (lanes 1, 3&11) *rif1* (lanes 6&8) and *rif2* (lanes 12&14) mutations increase the length of the telomeres considerably, with *rif1* mutants showing more pronounced effects. The double mutants have an additive effect and the telomeres are extremely long and disperse (lanes 17&19). Single copy expression of yeast Rif1 is able to complement *rif1* phenotype (lanes 5, 10, 16&21); however, dRif1 does not change the telomere length in any of these strains (wild type- lanes 2&4, *rif1*- lanes7&9, *rif2*- lanes 13&15 and *rif1rif2*- lanes 18&20). Apart from telomere length, yeast Rif1 has a negative regulation on telomere position effect (TPE). Therefore, we tested whether TPE in yeast is perturbed by dRif1 expression**.** However, dRif1 expression does not affect TPE either (Additional file 13). This suggests that the *Drosophila* protein does not retain much functional similarity to its yeast counterpart and therefore, cannot complement yRif1 in telomere maintenance. This is in contrast to hRif1, which increases telomere length in *rif2* mutants [17]. These data lead us to speculate that unlike hRif1, *Drosophila* Rif1, has lost the ability to interact with and interfere with telomere length regulation in yeast.

**Figure 7:**
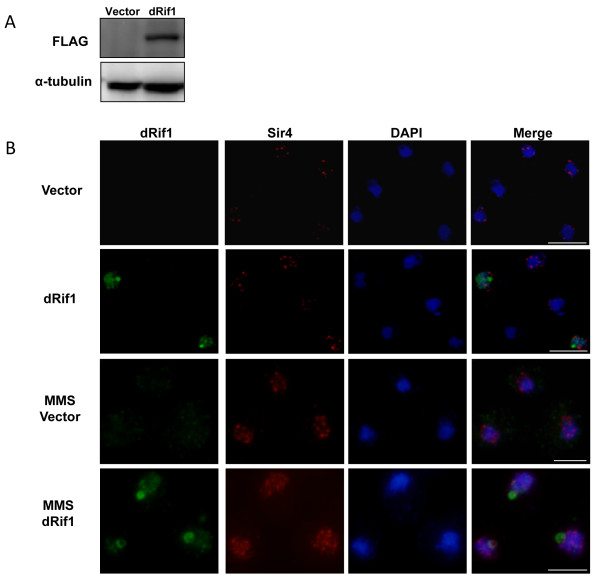
** A) FLAG tagged dRif1 is expressed in*S.cerevisiae.*** Total cell extracts of yeast transformed with empty vector (lane 1) or 3XFLAG tagged dRif1(lane 2) were probed with antibodies to FLAG and tubulin. Lanes 2 shows 3XFLAG dRif1 expression. **B)** dRif1 introduced in yeast localizes to the nucleus but not to yeast telomeres. Yeast cells transformed with empty vector or 3X FLAG dRif1 were stained for 3xFLAGdRif1 (green) and 13xmycSir4 (red). Nuclei were stained with DAPI (blue; Panel B, row 1&2). DNA damage induction with 0.05%MMS for 90 minutes treatment did not alter the localization of dRif1 in yeast (Panel B, rows 3,4). (Scale bar, 5 μm).

As a further test of functional conservation of dRif1, we determined the subcellular localization of dRif1 when expressed in yeast. We first confirmed by western blot analysis that dRif1 was expressed in yeast cells. As seen in Figure 7A, FLAG-tagged Rif1 could be detected in yeast. Empty vector or FLAG-tagged dRif1 was transformed into yeast strains that express myc-tagged Sir4 protein. Sir4, silent information regulator 4 protein, localizes to telomeric clusters and appears as 3 to 6 bright foci in the nuclei. These strains were fixed and immunofluorescence experiments were performed using FLAG (dRif1) and myc (Sir4) antibodies. We found that 20–30 percent of the cells have clear nuclear signal for dRif1, showing that dRif1 localizes to the nucleus in yeast (Figure 7B; panels 2 and 4). Empty vector transformed control cells did not show any signal for dRif1. However, there was no colocalization of the dRif1 with Sir4 protein. Therefore we conclude that dRif1 protein does not localize to the telomeres in yeast and this also explains the failure of dRif1 in complementing its yeast counterpart in our previous experiments. Since hRif1 has been shown to localize to aberrant, unprotected human telomeres, we tested dRif1 localization in *yku70* mutants as *yku70* mutant have damaged telomeres; but no relocalization of dRif1 to these sites could be detected (data not shown). There were two interesting features about dRif1 localization in yeast. First, only a subset seemed to show dRif1 staining, suggesting that not all cells were expressing dRif1. Second, dRif1 localized to a distinct compartment within the nucleus, which is neither telomeres, nor nucleolus. This novel site usually appeared as bright spot in the nucleus and sometimes appeared as a ring. Such novel site localization has recently been reported for Slx5, a component of the ubiqutin E3 ligase complex that targets sumoylated proteins and reported to have roles in DNA damage response [31]

Mammalian Rif1 is localized to the nucleus and relocalizes to the DNA damage/repair foci [16,17]. We induced DNA damage in yeast cells expressing dRif1 by incubating the overnight grown cultures with 0.05%MMS and checked for the dRif1 localization pattern upon DNA damage (Figure 7B, panels 3 and 4). We found that the staining remained the same and Rif1 retained its unique pattern; although now most of the nuclei showed the more prominent ring like localization unlike the prominent spot or small ring staining in the untreated cell nuclei. As reported, the Sir4p spots became more diffuse [32,33]. These data suggest that dRif1 does not relocalize to DNA damage sites in yeast as well.

### dRif1 does not co-localize with DNA damage sites in human cells

**Figure 8:**
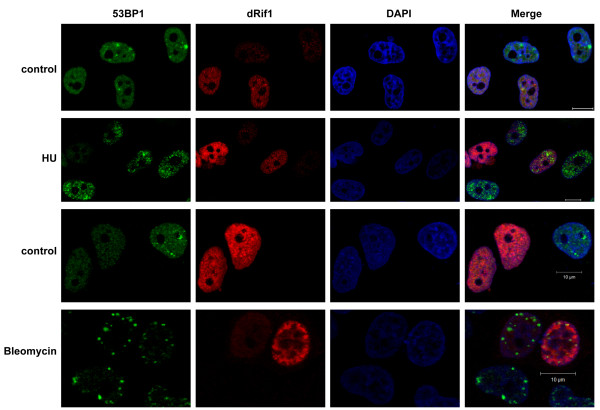
** Heterologous-expression of dRif1 in HeLa cells interferes with the 53BP1- foci formation upon DNA damage.** HeLa cells were transfected with full length dRif1 with a N-terminal 3XFLAG tag. Cells were either mock treated (row 1,3) or with 2 mM HU (row 2) or 50 μg/ml of bleomycin (row 4) for 4 hrs, fixed and stained for dRif1(red) and 53BP1 (green). DAPI (blue) was used to mark the nuclei. (Scale bar, 10 μm).

The lack of relocalization of dRif1 to sites of damage in yeast could be either due to lack of conservation of the partners or pathways in yeast or alternatively might indicate lack of conservation of this function in dRif1. In order to distinguish between the two possibilities, we expressed FLAG-dRif1 in HeLa cells. The full-length dRif1 along with 3XFLAG was cloned in pCMV vector and transfected into HeLa cells. Since dRIF1 colocalized with heterochromatin in S2 cells, we stained dRif1 using FLAG antibody to confirm transfection and also co-stained with H3K9Me3 antibody as heterochromatin marker. We did not find any significant colocalization of dRif1 with heterochromatin in HeLa cells (Additional file 14). After 24 hrs of transfection, the cells were treated with HU for 16 hrs or bleomycin for 4 hours and both treated and untreated cells were stained with antibodies to 53BP1 and FLAG. Untreated control cells showed clear nuclear localization of FLAG tagged dRif1 where as 53BP1 showed one or two foci (Figure 8, row1 and 3).

When HeLa cells were treated with HU (row2) or bleomycin (row4), we found that damage sites were marked with 53BP1. Eventhough hRif1 has been shown to accumulate at such damage foci, we observed that dRif1 did not accumulate in these sites [16-18]. An interesting feature of expressing dRif1 protein in HeLa cells was that when a larger amount of protein was expressed, the nuclei appeared deformed and additionally, did not show large 53BP1 spots or foci upon HU or bleomycin treatment. However, in cells not expressing dRif1, or expressing low levels of dRif1, prominent 53BP1 spots were observed, even though dRif1 did not colocalize with these damage spots, indirectly suggesting that dRif1 protein possibly interfered with the normal DNA damage response of HeLa cells. These data along with our previous result showing that dRif1 does not accumulate at DNA damage sites in *Drosophila* cells as well, suggest that the *Drosophila* homologue may not respond to DNA damage in the same manner as the human homologue.

## Discussion

Rif1 was identified in yeast almost two decades ago, and genetic and biochemical studies have clearly shown that it is a negative regulator of telomerase. Emerging evidence shows that Rif1 in mammals has diverged from its primary role in telomere synthesis and maintenance to a broader role in response to DNA damage. In this work we initiated a study on the *Drosophila* homologue of Rif1. Our detailed analysis of Rif1 from multiple organisms has identified several novel features. *Drosophila* Rif1 is evolutionarily closer to vertebrate Rif1 than yeast Rif1. All Rif1 homologues contain the conserved HEAT-repeats and this may carry out the core Rif1 activities. As this domain has been implicated in interacting with proteins, it might recruit a variety of proteins to carry out its functions. Within these HEAT repeats, our studies identify a conserved Rif1 specific repeat and this might be useful in identifying the core conserved interacting partners. The more diverse repeats are likely to facilitate participation in other functions. Our analysis also identified a conserved SILK motif, again present in all organisms, from yeast to humans. As this motif has been retained in all species, this is likely to participate in the core Rif1 functions. dRif1 lacks the C-terminal BLM interaction domain but contains all the conserved features associated with the N-terminal region.

dRif1 encodes for a 160 kDa protein that is localized to the nucleus. We find that a large fraction of the protein is associated with heterochromatin. In budding yeasts, yRif1 is predominantly associated with telomeric heterochromatin, although it is not required for establishment or maintenance of telomeric heterochromatin [9]. A very recent report implicates yRif1 in heterochromatin establishment at the silent mating type loci [34] and genome wide chromatin immunoprecipitation studies also show that yRif1 is associated with the silent mating type loci [11]. Rif1 in human cell lines were also shown to be associated with arrested replication forks in the vicinity of pericentromeric heterochromatin although this was not observed in unperturbed cells [18]. These results taken together implicate a possible evolutionarily conserved role for Rif1 at the heterochromatin.

We find that knock down of dRif1 does not lead to any difference in response to DNA damage in S2 cells suggesting that dRif1 is unlikely to function at repair sites. *Drosophila* and human Rif1 behave differently in yeast: whereas human Rif1 interferes with telomere length in yeast [17], *Drosophila* Rif1 does not. This suggests that human Rif1 has possibly retained its ability to interact with telomeric partners of Rif1, possibly Rap1, and *Drosophila* Rif1 has lost that ability perhaps, because unlike yeasts and vertebrates, *Drosophila* does not have Rap1. In this context, it is important to note that out of the 325 genomic targets identified for Rif1 in yeast, only about 88, mostly telomeric, colocalize with Rap1, suggesting there are a large number of Rap1 independent targets for Rif1 even in yeast [11]. We speculate that telomere independent functions of Rif1 are conserved in *Drosophila* and yeast and need to be explored.

Interestingly, upon DNA damage, dRif1 does not associate with the DNA repair foci although human Rif1 does. In fact, presence of dRif1 reduces the formation of DNA repair foci in HeLa cells. The C-terminus of vertebrate Rif1 has now been shown to interact with the BLM complex and also contain a DNA binding domain [20]. However, the *Drosophila* homologue does not have the extended C-terminus that carries out the critical functions of association with BLM protein. This suggests that *Drosophila* Rif1 may not have the ability to associate with replication forks and the differential response of dRif1 and human Rif1 to the presence of stalled replication forks are consistent with this.

The retention of Rif1 homologue in *Drosophila* raises an important question as to when and how Rif1 function diversified. As telomerase based telomere maintenance was replaced by alternate mechanisms of maintenance in many insects including *Drosophila*, telomerase and associated proteins have no counterparts in these organisms [35]. However, presence of Rif1 in *Drosophila* suggests that the recruitment of Rif1 to non-telomere based roles happened before *Drosophila* lost telomerase. Alternatively it might mean that Rif1 has both a telomeric and an evolutionarily conserved non-telomeric role in yeast. Even in yeast, only the C-terminus of Rif1 has been shown to interact with Rap1 and Rif2 proteins. The conserved N-terminal domain containing HEAT repeats has so far not been implicated in any function. Could this domain hold the clue to the evolutionarily conserved role of Rif1? Even though Rif1 was found as a negative regulator of telomerase, it has now been implicated in many more (previously unanticipated) functions like in telomere protection, recombination mediated telomere maintenance and repression of telomere specific transcripts [7,28,36]. However a molecular or biochemical basis underlying these functions is lacking. No specific motifs have been identified in Rif1 that could predict a biochemical function. Indeed there has been no structure –function analyses performed for any of the diverse Rif1 functions in yeast. Comparing the sequences of Rif1 throughout eukaryotes and experimental data obtained for Rif1 from the various model systems, it appears that the core conserved region of Rif1, the HEAT repeats and SILK motifs, warrant special attention. Studies in a genetically and developmentally tractable system like *Drosophila* will give us an additional important handle to understand the function of this conserved protein.

## Conclusion

In this study, we have carried out a detailed bioinformatic analysis of Rif1 and show that it is evolutionarily conserved across eukaryotes. Our study shows that within the HEAT repeats, there is a core Rif1 specific repeat region that is present in all the Rif1 homologues. A PP1 docking motif has also been identified in all Rif1 homologues. dRif1 is localized to the nucleus and shows a prominent heterochromatin association. It does not localize to foci induced by DNA damage. When tested for functional conservation of Rif1 function in dRif1, we find that it does not perturb or complement yeast Rif1 and does not relocalize to DNA damage foci in HeLa cells. The novel motifs identified in this study give a new perspective to investigate Rif1 functions, especially with respect to PP1 interactions and heterochromatin associations. Secondly, whether responding to DNA damage or binding to stalled replications forks is a newly acquired vertebrate specific function of Rif1 needs to be examined. This would suggest a further evolution and sub-functionalization of an ancient protein.

## Methods

### Bioinformatics methods

Rif1 protein sequence of human and yeast were used for finding the homologues in NCBI protein sequence database using PSI BLAST [37]. After three rounds of iteration, irrespective of their percentage of sequence homology, all the sequences were considered as putative homologues and subjected to motif prediction by MEME tool [38]. The motifs which are common between human and yeast Rif1 were considered as conserved domains across the homologues. HMM profiles were generated for the motifs using HMMer tool and was used to mine the NCBI protein sequence database [39]. This reverse profile based search strategy was helpful for us to mine the true homologue. The multiple sequence alignment of protein and motif sequences was done using ClustalW and ClustalX [40,41]. The Rif1 homologues were subjected for phylogenetic analysis using ClustalX neighbour joining algorithm [40]. The tree was constructed with 1000 replicates with a random bootstrap value. The consensus tree was visualized using MEGA software [42]. A simple pattern search program was written in Perl to identify the core conserved residues of PP1 interaction domain/SILK motifs and the search was done in the NCBI protein sequence build for human, *Drosophila* and yeast.

### Cloning and expression of dRif1

Full-length cDNA clone of CG30085-dRif1 (RE66338) was obtained from the Drosophila Genomics Resource Center (DGRC). In order to tag the dRif1 protein, we designed an oligonucleotide encoding 3X FLAG tag with sequence for NcoI compatible over hang at both the ends, annealed to get double stranded DNA, and cloned in frame at the start of the dRif1 in RE66338. The sequence and orientation of the tag was confirmed by DNA sequencing. To express the protein in yeast, we inserted the full length 3xFLAG tagged construct in pRS315 and yEpLac-181 vectors under the control of the yeast Rif1 promoter. These plasmids were transformed into yeast strain (KRY-109) containing a Sir413xMyc. The same 3xFLAG tagged full length protein was transferred to pCMV vector for HeLa cell experiments.

### Yeast transformation and telomere blots

Yeast transformation was done using lithium acetate mediated transformation and DNA was isolated using zymolyase. Genomic DNA was isolated from all the strains (wild type, *rif1, rif2 and rif1rif2* mutants expressing empty vector, yRif1 or dRif1). Approximately 1.5 μg of genomic DNA was digested with Xhol and subjected to electrophoresis on a 0.8% agarose gel along with 1 kb ladder (New England Biolabs). The gel was soaked in 0.4 N NaOH for 10 min, and capillary transferred to charged Nylon membrane (IMMOBILON-NY+, Millipore) using 0.4 N NaOH. The membrane was hybridized to the radiolabelled dGT/CA repeat at 55°C. XhoI cuts yeast genomic DNA at a conserved site that is 1.5 kb upstream of the telomere repeats and when probed with telomere sequence, the size of this band indicates the length of the telomere.

### Immunofluorescence

Yeast cells were grown in SC-Leu selective media overnight and the cells were fixed with formaldehyde, spheroplasted and spotted on a poly-lysine coated multi-well slide. It was then treated with pre-chilled methanol and acetone for further permeabilization. The cells were then blocked in PBST (0.1% triton X100) containing 0.1%BSA and incubated with appropriately diluted primary antibodies in PBST overnight at 4°C. Washes were performed to remove unbound antibodies and incubated with secondary antibodies conjugated with fluorophores, washed again and mounted with mounting media containing DAPI. In yeast, DNA damage was induced by treating the cells with 0.05% of MMS (SIGMA) for 90minutes. After that the cells were harvested and immunofluorescence performed as described before and the images were captured in an Olympus IX81 microscope. For HeLa and S2 cells, cells were plated and grown on cover slips. They were formaldehyde fixed, permeabilized, blocked and stained using antibodies indicated at appropriate dilutions. The slides were imaged in a multiphoton LSM-510 Zeiss confocal or LSM-710 Zeiss confocal microscope. Antibodies used in the study are H2AvD pS137 (Rockland Immunochemicals), 53BP1 (Santacruz), Myc (Abcam) and FLAG-M2 (Sigma). Polyclonal antibodies to dRif1 were raised in rabbit. Amino acids 694–1094 of dRif1 was expressed in bacteria as a 6x-HIS tagged fusion protein; purified and immunized a rabbit for antibody production. The serum was affinity purified against the same bacterially expressed protein bound to nitrocellulose membrane before use.

### Knock down of dRif1 in S2 cells

Double stranded RNA (dsRNA) was used to knock-down dRif1 levels in S2 cells. Three different primer sets were designed (with no off target) along with 5′ T7 binding site. Primer sequences will be provided upon request. GFP dsRNA was used as control/mock experiment. MEGAscript T7 kit from Ambion was used to make dsRNA according to the manufacturer’s instructions and checked on gel for integrity of RNA made and stored at -20°C until use. 1X10^6^ cells/ml of S2 cells were treated with ~30 μg of the dsRNA in serum free media for 30 minutes and later supplemented with serum containing media. After 4 days one more round of dsRNA treatment was given to completely knockdown dRif1. These cells were then processed for RNA isolation, IF and DNA damage induction treatment. 2.5 mM HU for 16 hrs, 50 μg/ml bleomycin for 4 hrs were used for the DNA damage induction. Cells were harvested and total protein and RNA were made from untreated and treated samples. A fraction of the same sets of cells were processed for immunofluorescence.

### RNA isolation and RT PCR

RNA isolation was carried out using trizol method and treated with RNase free DNase. 2 μg of RNA was used to make cDNA and to check the telomeric transcription levels. Telomeric transcription was assessed by comparative C_T_ method in ABI 7500FAST machine using SYBER GREEN chemistry using primers mentioned below [30,43]. Signals were normalized against Rps17 as internal control. The telomeric transcript levels of dRif1 RNA treated samples were compared against GFP RNA treated samples (set as 100). Graphs were plotted with relative level of telomeric transcription for each locus. Averages are from two independent experiments and error bars indicate standard deviation. Sequence of primers used to detect the telomeric and control transcripts are mentioned below.

RpS17-F→AAGCGCATCTGCGAGGAG

RpS17-R→CCTCCTCCTGCAACTTGATG

HeT-F→TTGTCTTCTCCTCCGTCCACC

HeT-R→GAGCTGAGATTTTTCTCTATGCTACTG

TAHRE-F→CTTCCCCTCCGCTCTCATC

TAHARE-R→CCTAGATCTGCATTTGTATTAGTAGCTG

TART-F→CAAAAAATCCTTTCCGAGATCC

TART-R→GGGCATCAATATTTAGAATGAACAG

Jockey-F→ACGACTCAATCTAGGGCTCGTG

Jockey-R→CGTCCATTCTCGTATTGATGG

## Competing interests

The authors declare that they have no competing interests.

## Authors’ Contributions

KM conceived the study, KM and RM designed the study, RS performed the bioinformatic analysis, ES performed the experiments, VB performed experiments with flies, KM, ES, RS and RM analysed the data, and KM wrote the paper. All authors read and approved the final manuscript.

## Supplementary Material

Additional file 1 **The list of Rif1 homologues**. The organism name, common name and the NCBI accession number of the Rif1 homologues are given in the table. Click here for file

Additional file 2 **Expanded Phylogenetic tree of Rif1 homologues**. The consensus phylogenetic tree of Rif1 homologues drawn by the neighbour joining method is shown. The random sampling was done for 1000 replicates and the branches having bootstrap value above 50 percentage are shown in the figure. The corresponding protein sequence accession number for the organisms mentioned in the tree is given the Additional file 1. Click here for file

Additional file 3 **Putative plant homologues of Rif1**. The organism name, common name and the NCBI accession number of the Rif1 homologues are given in the table. Click here for file

Additional file 4 **The core conserved region of HEAT repeat.** The organism name and the length of the domain for each sequence are shown to the left and right of the multiple sequence alignment, respectively. The amino acids are highlighted in different colours based on their property. The degree of conservation at each position in the alignment is represented as bar graph at the bottom of the alignment. Click here for file

Additional file 5 **The N-terminal SILK/PP1 interaction domain of unicellular organisms**. The organism name and the length of the domain for each sequence are shown to the left and right of the multiple sequence alignment, respectively. The amino acids are highlighted in different colours based on their property. The degree of conservation at each position in the alignment is represented as bar graph at the bottom of the alignment. Click here for file

Additional file 6 **The C-terminal SILK/PP1 interaction domain of multicellular organisms**. The organism name and the length of the domain for each sequence are shown to the left and right of the multiple sequence alignment, respectively. The amino acids are highlighted in different colors based on their property. The degree of conservation at each position in the alignment is represented as bar graph at the bottom of the alignment. Click here for file

Additional file 7 **The domain shift relationship among organisms that have Rif1.** The organisms having N-terminal or C-terminal SILK/PP1 interaction domain are highlighted in red and green, respectively in the tree. Click here for file

Additional file 8 **The list of proteins with SILK/PP1 interaction domain in*Homo sapiens***. The NCBI accession number, protein name, SILK/PP1 interaction domain, protein size, domain length and the position of the motif for the proteins having SILK/PP1 interaction domain are listed in the table. Click here for file

Additional file 9 **The list of proteins with SILK/PP1 interaction domain in*Drosophila melanogaster***. The NCBI accession number, protein name, SILK/PP1 interaction domain, protein size, domain length and the position of the motif for the proteins with SILK/PP1 interaction domain are listed in the table. Click here for file

Additional file 10 **The list of proteins with SILK/PP1 interaction domain in*Saccharomyces cerevisiae***. The NCBI accession number, protein name, SILK/PP1 interaction domain, protein size, domain length and the position of the motif for the proteins with SILK/PP1 interaction domain are listed in the table. Click here for file

Additional file 11 **The DNA binding domain of hRif1 is conserved across the homologues**. The organism name and the length of the domain for each sequence are shown to the left and right of the multiple sequence alignment, respectively. The amino acids are highlighted in different colours based on their property. The degree of conservation at each position in the alignment is represented as bar graph at the bottom of the alignment. Click here for file

Additional file 12 **The BLM1 interaction domain of hRif1 is conserved across vertebrates**. The organism name and the length of the domain for each sequence are shown to the left and right of the multiple sequence alignment, respectively. The amino acids are highlighted in different colours based on their property. The degree of conservation at each position in the alignment is represented as bar graph at the bottom of the alignment. Click here for file

Additional file 13 **Telomere position effect is not altered by expression of dRif1 in yeast.** Wild type, *rif1*, *rif2* and *rif1rif2* strains were transformed with empty vectors (pRS315, yEPLac181), dRif1 (pRS315dRif1, yEPLac181dRif1) and yeast Rif1 (pRS315yRif1). All strains contain *URA3* gene at the telomere of chromosome VIIL. y*ku70* mutant is a positive control for loss of gene silencing; *URA3* is expressed and therefore not growing on FOA plate. The silencing on FOA plates with tenfold dilution and spotting assay do not show difference in growth compared to the corresponding vector alone control in wild type, *rif1*, *rif2* and *rif1rif2* double mutant strains. Click here for file

Additional file 14 **dRif1 does not significantly colocalize with H3K9Me**_**3**_**in HeLa cells.** HeLa cell transfected with FLAG-dRif1 were stained with anti-FLAG antibody (red) and H3K9Me3 antibody (green). DAPI is seen as blue staining. Click here for file
