# Supplementary material for: Functional diversification of yeast telomere associated protein, Rif1, in higher eukaryotes
Source: BMC Genomics. 2012 Jun 19;13:255. doi: 10.1186/1471-2164-13-255 (PMC3410773; doi:10.1186/1471-2164-13-255)
Supplement: Additional file 1 — The list of Rif1 homologues. The organism name, common name and the NCBI accession number of the Rif1 homologues are given in the table. [file 1471-2164-13-255-S1.pdf]

Additional file 1. Rif1 homologues in different organisms

Organism	Common name	NCBI accession number
Ajellomyces capsulatus G186AR	Darling's disease fungus	EEH07350.1
Ajellomyces dermatitidis SLH14081	Tropical fungus	XP_002629282.1
Arthroderma benhamiae CBS 112371	fungus	XP_003015601.1
Arthroderma gypseum CBS 118893	fungus	XP_003169470.1
Arthroderma otae CBS 113480	fungus	XP_002849712.1
Ashbya gossypii ATCC 10895	fungus	NP_984675.1
Aspergillus clavatus NRRL 1	fungus	XP_001271779.1
Aspergillus flavus NRRL3357	fungus	XP_002376949.1
Aspergillus fumigatus Af293	fungus	XP_751941.1
Aspergillus nidulans FGSC A4	fungus	XP_659264.1
Aspergillus niger CBS 513.88	fungus	XP_001401739.1
Aspergillus oryzae RIB40	fungus	XP_001821189.2
Aspergillus terreus NIH2624	fungus	XP_001214475.1
Botryotinia fuckeliana B05.10	Fungus plant pathogen	XP_001561183.1
Candida albicans WO-1	fungus	EEQ42644.1
Candida dubliniensis CD36	fungus	XP_002417177.1
Candida glabrata	fungus	AAT67386.1
Candida tropicalis MYA-3404	fungus	XP_002550236.1
Chaetomium globosum CBS 148.51	Soil fungus	XP_001220993.1
Clavispora lusitaniae ATCC 42720	Candida lusitaniae	XP_002618754.1
Coccidioides immitis RS	fungus	XP_001241144.1
Coccidioides posadasii C735 delta SOWgp	fungus	XP_003065092.1
Debaryomyces hansenii CBS767	Salt tolarent yeasts	XP_002769956.1
Gibberella zeae PH-1	Fungus plant pathogen	XP_381686.1
Glomerella graminicola M1.001	fungus	EFQ27809.1
Kluyveromyces lactis NRRL Y-1140	yeast	XP_455136.1
Lachancea thermotolerans	yeast	XP_002556318.1
Leptosphaeria maculans	Fungus plant pathogen	CBY00494.1
Lodderomyces elongisporus NRRL YB-4239	yeast	XP_001526918.1
Magnaporthe oryzae 70-15	rice blast fungus	XP_362839.2
Nectria haematococca mpVI 77-13-4	Fungus pathogen	XP_003049857.1
Neosartorya fischeri NRRL 181	Fungus pathogen	XP_001267138.1
Neurospora crassa OR74A	Fungus	XP_963945.1
Paracoccidioides brasiliensis Pb01	pathogenic fungus	XP_002794700.1
Penicillium chrysogenum Wisconsin 54-1255	Fungus	XP_002562147.1
Penicillium marneffei ATCC 18224	Fungus	XP_002150486.1

Phaeosphaeria nodorum SN15	Glume blotch fungus	XP_001797000.1
Pichia pastoris GS115	methylotrophic yeast	XP_002489801.1
Podospora anserina S mat+	Fungus	XP_001912591.1
Pyrenophora teres f. teres 0-1	Fungus plant pathogen	EFQ93104.1
Pyrenophora tritici-repentis Pt-1C-BFP	Fungus plant pathogen	XP_001931945.1
Saccharomyces cerevisiae S288c	Yeast	NP_009834.1
Scheffersomyces stipitis CBS 6054	Fungus	XP_001387854.2
Schizosaccharomyces japonicus yFS275	Fungus	XP_002172627.1
Schizosaccharomyces pombe 972h-	Fungus	NP_593910.2
Sclerotinia sclerotiorum 1980	Fungus plant pathogen	XP_001594372.1
Sordaria macrospora	fruit body Fungus	CBI56133.1
Talaromyces stipitatus ATCC 10500	fungus	XP_002483707.1
Trichophyton verrucosum HKI 0517	fungus	XP_003025698.1
Tuber melanosporum Mel28	fruit body Fungus	XP_002837628.1
Uncinocarpus reesii 1704	fungus	XP_002542972.1
Vanderwaltozyma polyspora DSM 70294	fungus	XP_001645061.1
Verticillium albo-atrum VaMs.102	Fungus plant pathogen	XP_003002595.1*
Zygosaccharomyces rouxii	yeast	XP_002495349.1
Aedes aegypti	yellow fever mosquito	XP_001653011.1
Anopheles darlingi	mosquito	EFR21139.1
Anopheles gambiae str. PEST	mosquito	XP_001230813.2
Camponotus floridanus	ant species	EFN73980.1
Drosophila ananassae	Fruitfly species	XP_001960452.1
Drosophila erecta	Fruitfly species	XP_001975459.1
Drosophila grimshawi	Fruitfly species	XP_001986289.1
Drosophila melanogaster	Fruitfly species	NP_725497.1
Drosophila persimilis	Fruitfly species	XP_002018287.1
Drosophila pseudoobscura pseudoobscura	Fruitfly species	XP_001361126.1
Drosophila sechellia	Fruitfly species	XP_002034026.1
Drosophila simulans	Fruitfly species	XP_002081674.1
Drosophila virilis	Fruitfly species	XP_002050586.1
Drosophila willistoni	Fruitfly species	XP_002061311.1
Harpegnathos saltator	Jerdon's jumping ant	EFN87937.1
Nasonia vitripennis	jewel wasps	XP_001605319.1
Tribolium castaneum	Red flour beetle	EFA10163.1
Ixodes scapularis	deer tick or blacklegged tick	XP_002406456.1
Ailuropoda melanoleuca	giant panda	EFB21675.1
Bos taurus	Cow	XP_615269.3
Callithrix jacchus	New World monkey	XP_002749499.1

Danio rerio	Zebrafish	CAQ15348.1
Equus caballus	Horse	XP_001491023.2
Gallus gallus	Chicken	XP_422162.2
Homo sapiens	Human	NP_060621.3
Macaca mulatta	monkey	XP_002808045.1
Mus musculus	Mouse	NP_780447.4
Oryctolagus cuniculus	European Rabbit	XP_002712208.1
Pan troglodytes	Chimp	XP_001137423.1
Pongo abelii	Sumatran Orangutan	XP_002812524.1
Rattus norvegicus	Rat	XP_215736.5
Sus scrofa	wild pig	XP_003133872.1
Taeniopygia guttata	Zebra Finch (bird)	XP_002188647.1
Xenopus (Silurana) tropicalis	Western clawed frog	XP_002936710.1
Saccoglossus kowalevskii	acorn worm (Hemi chordata)	XP_002739208.1*
Selaginella moellendorffii	Lycopodiophyta (vascular Plant)	XP_002967836.1*
Trichoplax adhaerens	Placozoa, basal group of multicellular animals	XP_002110766.1
Hydra magnipapillata	water polyp Cnidarian	XP_002154730.1
