# Supplementary figures and images for: Functional diversification of yeast telomere associated protein, Rif1, in higher eukaryotes
Source: BMC Genomics. 2012 Jun 19;13:255. doi: 10.1186/1471-2164-13-255 (PMC3410773; doi:10.1186/1471-2164-13-255)

Additional file 2

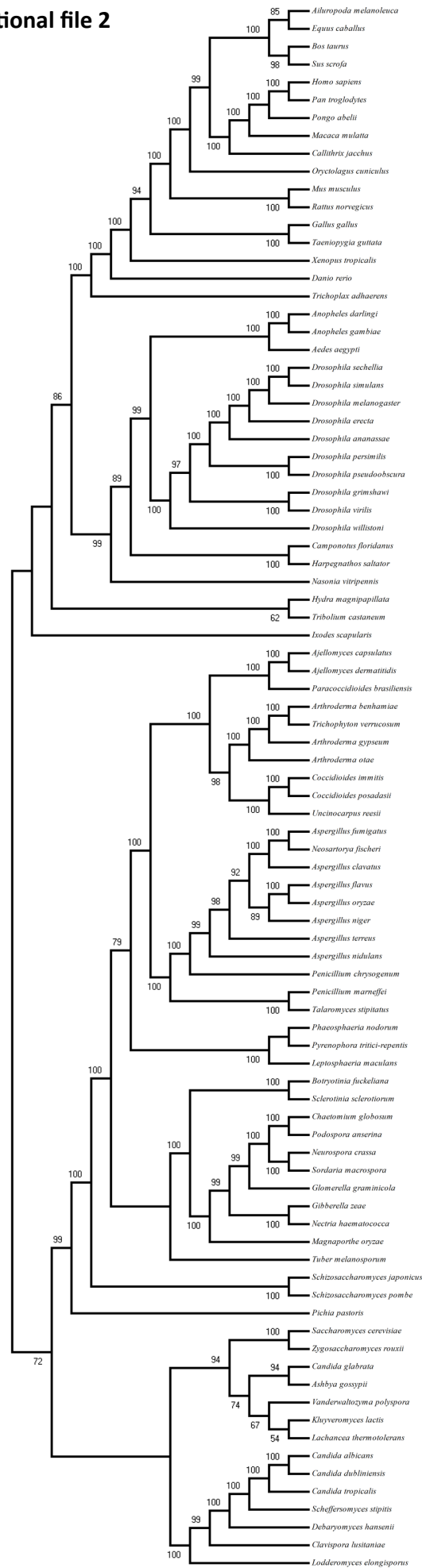

Supplement: Additional file 2 — Expanded Phylogenetic tree of Rif1 homologues. The consensus phylogenetic tree of Rif1 homologues drawn by the neighbour joining method is shown. The random sampling was done for 1000 replicates and the branches having bootstrap value above 50 percentage are shown in the figure. The corresponding protein sequence accession number for the organisms mentioned in the tree is given the Additional file 1. [file 1471-2164-13-255-S2.pdf]

Additional file 7

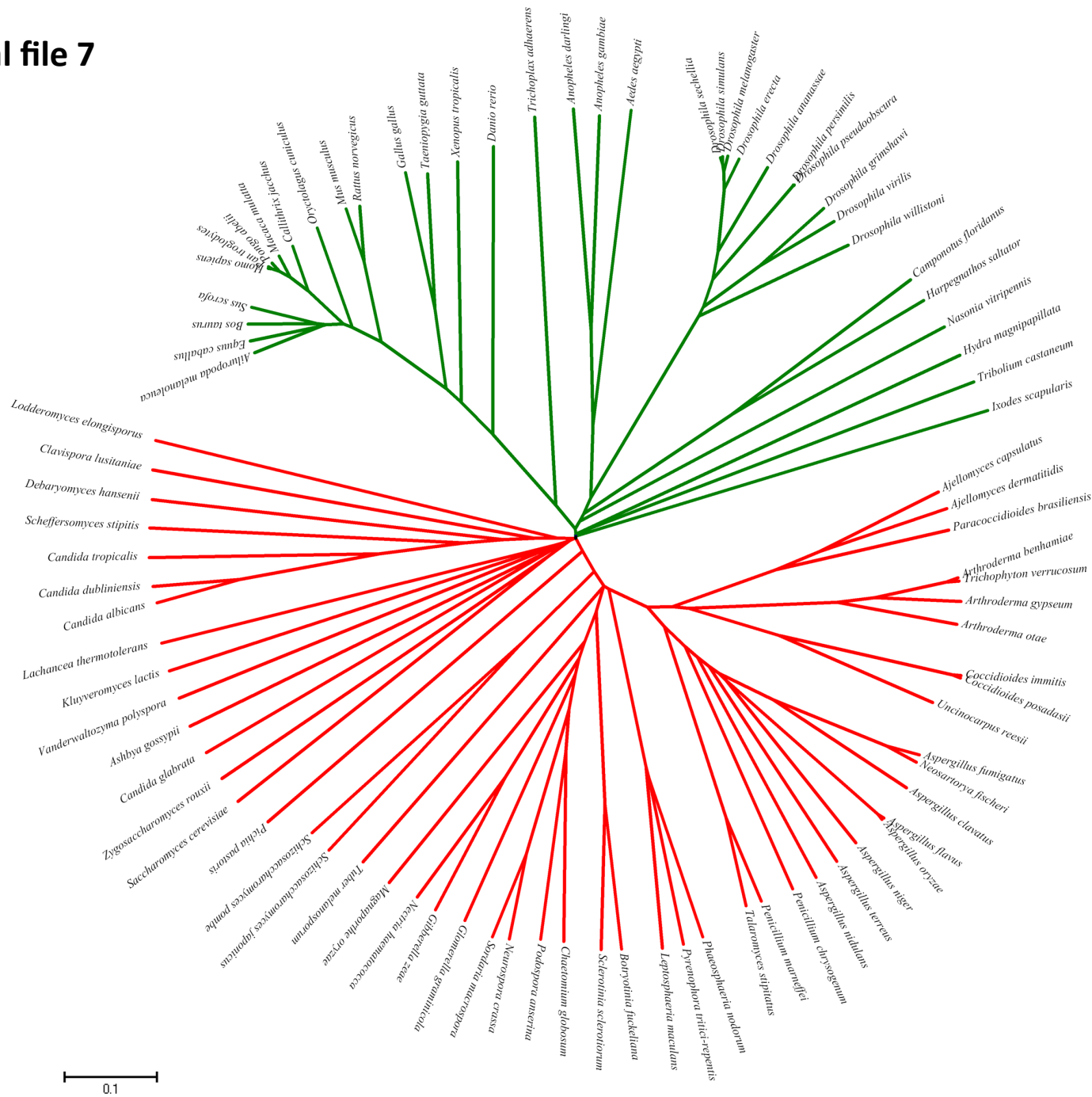

Supplement: Additional file 7 — The domain shift relationship among organisms that have Rif1. The organisms having N-terminal or C-terminal SILK/PP1 interaction domain are highlighted in red and green, respectively in the tree. [file 1471-2164-13-255-S7.pdf]

# Additional file 12

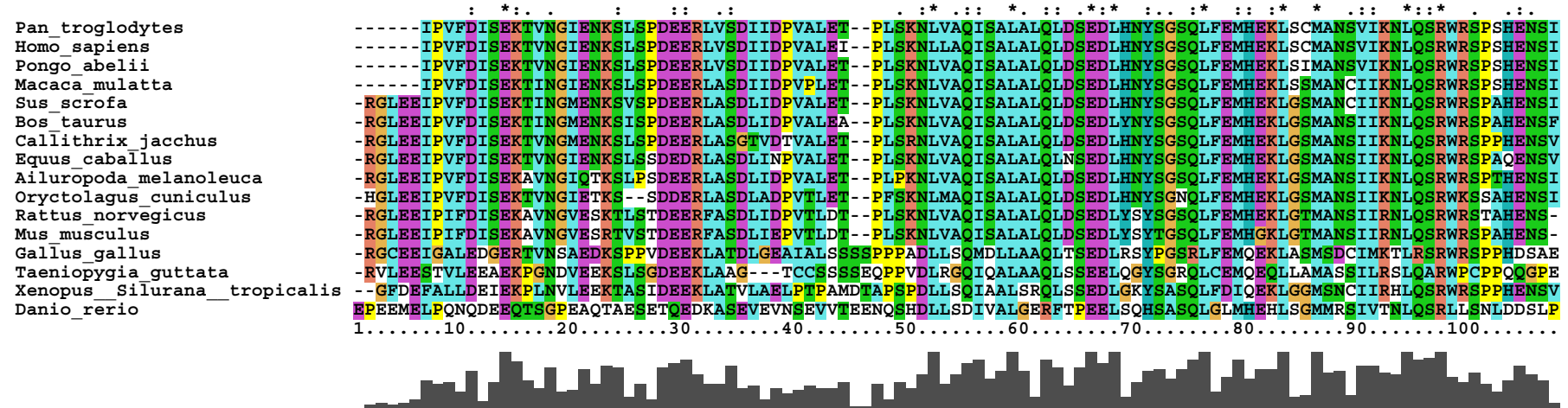

Supplement: Additional file 12 — The BLM1 interaction domain of hRif1 is conserved across vertebrates. The organism name and the length of the domain for each sequence are shown to the left and right of the multiple sequence alignment, respectively. The amino acids are highlighted in different colours based on their property. The degree of conservation at each position in the alignment is represented as bar graph at the bottom of the alignment. [file 1471-2164-13-255-S12.pdf]

Additional file 13

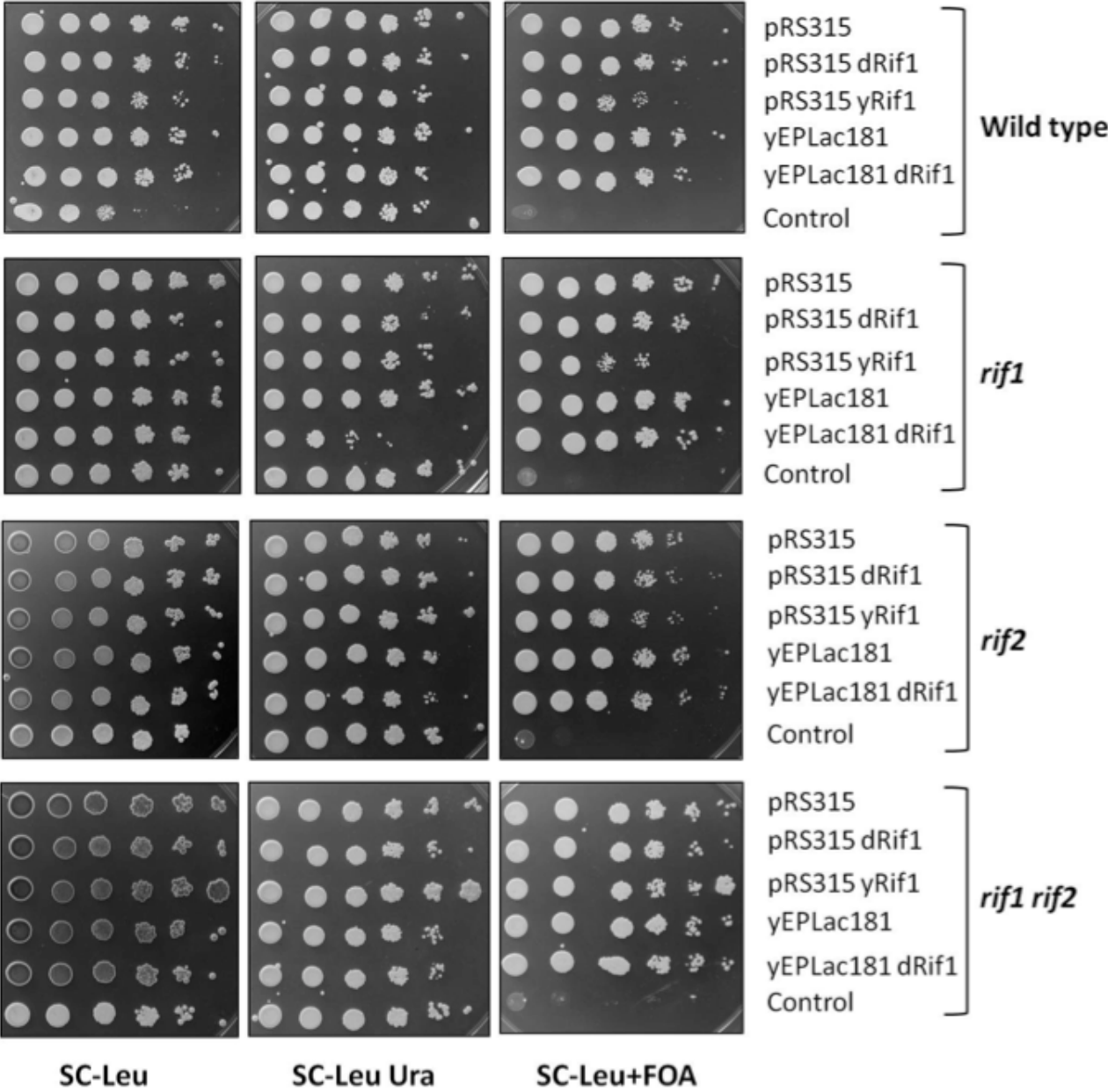

Supplement: Additional file 13 — Telomere position effect is not altered by expression of dRif1 in yeast. Wild type, rif1, rif2 and rif1rif2 strains were transformed with empty vectors (pRS315, yEPLac181), dRif1 (pRS315dRif1, yEPLac181dRif1) and yeast Rif1 (pRS315yRif1). All strains contain URA3 gene at the telomere of chromosome VIIL. yku70 mutant is a positive control for loss of gene silencing; URA3 is expressed and therefore not growing on FOA plate. The silencing on FOA plates with tenfold dilution and spotting assay do not show difference in growth compared to the corresponding vector alone control in wild type, rif1, rif2 and rif1rif2 double mutant strains. [file 1471-2164-13-255-S13.pdf]

Additional file 14

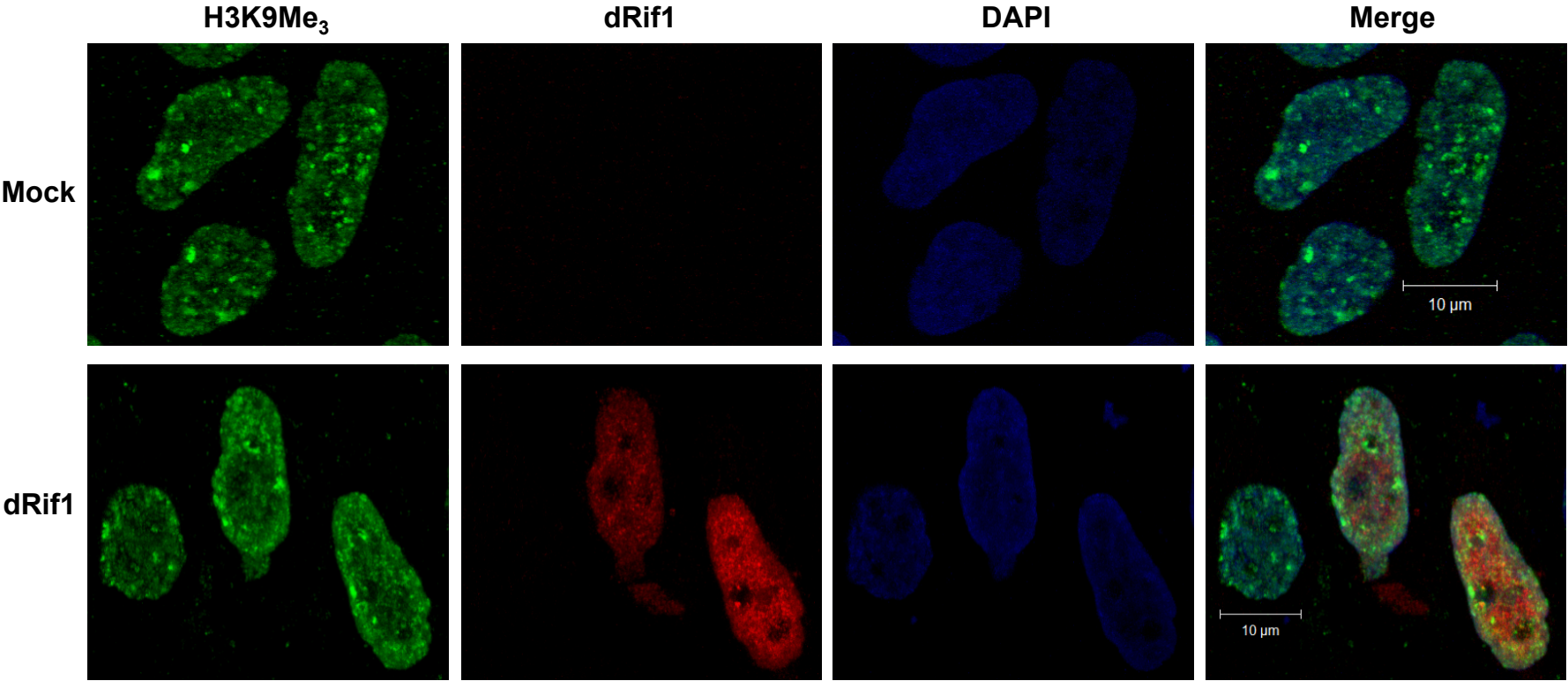

Supplement: Additional file 14 — dRif1 does not significantly colocalize with H3K9Me3in HeLa cells. HeLa cell transfected with FLAG-dRif1 were stained with anti-FLAG antibody (red) and H3K9Me3 antibody (green). DAPI is seen as blue staining. [file 1471-2164-13-255-S14.pdf]
