# Supplementary material for: Functional diversification of yeast telomere associated protein, Rif1, in higher eukaryotes
Source: BMC Genomics. 2012 Jun 19;13:255. doi: 10.1186/1471-2164-13-255 (PMC3410773; doi:10.1186/1471-2164-13-255)
Supplement: Additional file 3 — Putative plant homologues of Rif1. The organism name, common name and the NCBI accession number of the Rif1 homologues are given in the table. [file 1471-2164-13-255-S3.pdf]

**Additional file 3. Putative plant homologues of Rif1**

| Organism | Common name | NCBI accession number |
|---|---|---|
| *Vitis vinifera* | Grape Vine | XP_002282389.1, CAN64432.1 |
| *Populus trichocarpa* | black cottonwood | XP_002325200.1 |
| *Ricinus communis* | castor oil plant | XP_002531956.1 |
| *Oryza sativa* | Rice | EAY99866, BAD72285.1 |
| *Sorghum bicolor* | sorghum | XP_002436572.1 |
