# Supplementary material for: Functional diversification of yeast telomere associated protein, Rif1, in higher eukaryotes
Source: BMC Genomics. 2012 Jun 19;13:255. doi: 10.1186/1471-2164-13-255 (PMC3410773; doi:10.1186/1471-2164-13-255)

Additional file 4

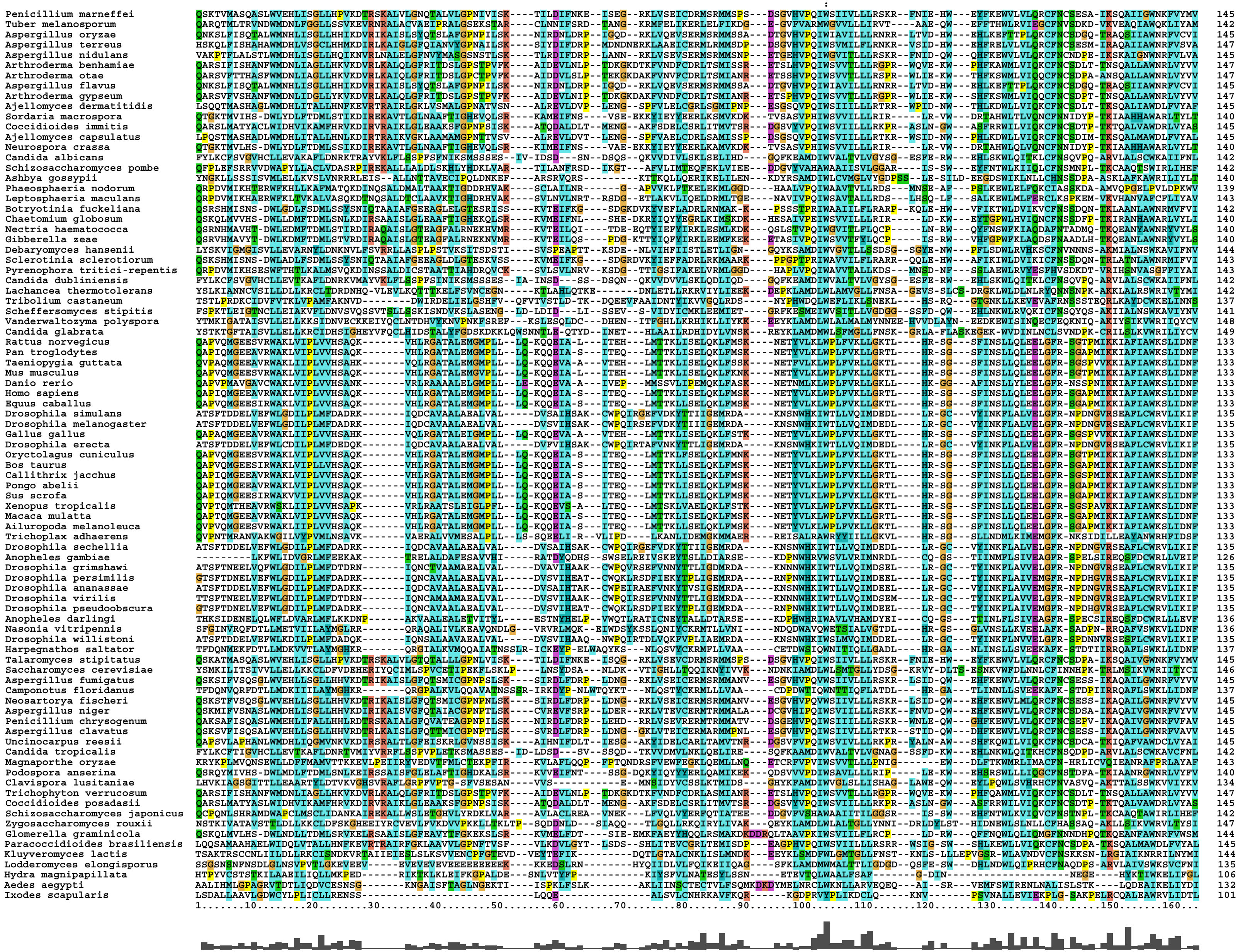

Supplement: Additional file 4 — The core conserved region of HEAT repeat. The organism name and the length of the domain for each sequence are shown to the left and right of the multiple sequence alignment, respectively. The amino acids are highlighted in different colours based on their property. The degree of conservation at each position in the alignment is represented as bar graph at the bottom of the alignment. [file 1471-2164-13-255-S4.pdf]
