# Supplementary material for: Functional diversification of yeast telomere associated protein, Rif1, in higher eukaryotes
Source: BMC Genomics. 2012 Jun 19;13:255. doi: 10.1186/1471-2164-13-255 (PMC3410773; doi:10.1186/1471-2164-13-255)

Additional file 5

```
                               * * :                                                 **:
Schizosaccharomyces japonicus  KRVVFSPHPDN---SP--------------GGFLPKHG----------IP-KKSILK   29
Schizosaccharomyces pombe      KKVNFSSELEN---SP--------------GGNRPSFG----------LP-KRGILK   29
Paracoccidioides brasiliensis  KRVNFSPLTSY-IKPPSFTNPNNPKTRTPLRALLPS---------NQYKP-SKSILK   46
Coccidioides immitis           KRVNFSPLTSY-IKAPEFPASLAKNSRPPLRSLRPS---------KQCKP-AKSILK   46
Coccidioides posadasii         KRVNFSPLTSY-IKAPEFPASLAKNSRPPLPSLRPS---------KQCKP-AKSILK   46
Uncinocarpus reesii            KRVNFSPLTSY-IRTPDISSSLAK-TRCHLRSLRPS---------KQCKP-AKSILK   45
Arthroderma benhamiae          KRVNFSPMASY-IKPPEFT-DHSMKPPTRLRSLPPSS--------NQSVP-AKSILK   46
Trichophyton verrucosum        KRVNFSPMASY-IKPPEFT-DHSMKPPTRLRSLPPSS--------NQSVP-AKSILK   46
Arthroderma gypseum            KRVNFSPMASY-IKPPEFT-DHSMKSLIRLRSLPPSS--------NQSVP-AKSILK   46
Arthroderma otae               KRVNFSPMASY-IKPPELT-DYSTKSYVRLRSLPPSS--------SQSVP-AKSILK   46
Tuber melanosporum             KRVGWSPWTKY-HK-P-D-SLLS---VTPIRSAIDP------------KS-LKSILK   37
Chaetomium globosum            KKVGFSAQAEY-KEPPVYPDGEGVRQHP--TPVSMP-R-------SASKP-VKSILK   45
Podospora anserina             KKVGFSAQTEY-KEAPVYGEGGAVKQHP--TPVSLP-R-------SASKP-IKSILK   45
Neurospora crassa              KRVGFSAKAEY-KDAPVFIDG-DKRQQP--TPVSLP-R-------SASKP-VKSILK   44
Sordaria macrospora            KRVGFSAKAEY-KDAPVFIDG-DKRQQP--TPVSLS-R-------RASKP-VKSILK   44
Magnaporthe oryzae             KKVEFSAKTEY-RE-PLEYQSNEDVRRQR-TPASIPHR-------SAP---VKSILK   44
Glomerella graminicola         KRVGFSAKAQY-NEAPHYQG-VNVSGKR--SPLSVPSS-------TQPKP-VKGILK   45
Botryotinia fuckeliana         KKVVWSEKYDY-KDPPKIITDRKTLSA-VLSPLTP--S-------AERKA-TKSILK   45
Sclerotinia sclerotiorum       KKVGWSEKYDY-KDPPKIVTDKHILSAHVISPLTP--S-------AERKA-TKSILK   46
Gibberella zeae                KKVEWSSHTEY-KDPPD-LETSRFFKS---SPITTPSA-------ASSRP-IKGILK   44
Nectria haematococca           KKVEWSSHTEY-KEPPDYRDLTKYFRS---SPLSAPSS-------ASSKP-VKGILK   45
Phaeosphaeria nodorum          KRVNFELQLCT---TPHKKAIARSWTPSRSSPLRPLPQT------RVSRP-LKSILK   47
Pyrenophora tritici-repentis   KRVNFEL--CD---IPNRTAIAHSWTPTRSSPLRPLPQT------RLIQP-LKSILK   45
Leptosphaeria maculans         KRVNFELQTCA---IPTNNALTKDWTPTRSSPLRPLPQT------RVSLP-LKSILK   47
Aspergillus clavatus           KRVNFSPWTKY-IKPPSFANATLK-SKSELKALPPS---------NECKP-AKSILK   45
Aspergillus flavus             KRVNFSPWPK---SHP---N------KSDLKALPPS---------NECKP-SKSILK   35
Aspergillus oryzae             KRVNFSPWPK---SHP---N------KSDLKALPPS---------NECKP-SKSILK   35
Aspergillus niger              KRVNFSPWTRY-IKPPSFTNSAAK-SKSDLKSLPPS---------NECKP-TKSILK   45
Aspergillus terreus            KRVNFSPWTRY-IKPPSFTDPAAT-PEPELKALPPS---------NECKP-AKSILK   45
Penicillium marneffei          KKVNFSPITSY-IKPPTFSSRASM-SSNEVRPIPPS---------NECKP-AKSILK   45
Talaromyces stipitatus         KKVNFSPITNY-IKPPTFSSRASM-SSNEVRPIPPS---------NECKP-AKSILK   45
Penicillium chrysogenum        KKVNFSPWPKY-IKPPTFAS-AMK-SAPDVKTIPPS---------PNSKP-TKSILK   44
Ajellomyces capsulatus         KRVNFSPLTSY-IKPPSFPTSNAHKSRTPLRALPPS---------NQCKP-SKSILK   46
Ajellomyces dermatitidis       KRVNFSPMTSY-IKPPSFASSNSQKSRTPLRALPPS---------NQCKP-SKSILK   46
Aspergillus fumigatus          KRVNFSPWTKY-IKPPSFTNSAPR-LKSELKELPPS---------NECKP-TKSILK   45
Neosartorya fischeri           KRVNFSPWTKY-IKPPSFTNSALR-LKSELKELPPS---------NECKP-TKSILK   45
Aspergillus nidulans           KRVNFSPWPKF-IKPPSFTN-----PKS--KALLPS---------NDLKP-VRSILK   39
Candida glabrata               KSVSFLDESNK--NSEST-----------SSN--SS------------KP-KKSILR   29
Saccharomyces cerevisiae       KSVAFSDRIES---SPIYRIPG-------SSP-KPSPS---------SKP-GKSILR   36
Vanderwaltozyma polyspora      KSVAFSDKIES---SPTNKDPL-------HTPSRPSSI---------TKP-KRSILR   37
Zygosaccharomyces rouxii       KTVAFSDRVES---SPTQQTPR-------SSP-RPSSL---------QKP-VKSILR   36
Kluyveromyces lactis           KGVSFSDRVES---SPTMQTMG-------SSPIRPSSM---------TKPPARSILK   38
Lachancea thermotolerans       KSVAFSDQIDS---SPPPASLH-------SSP-HRSSQ---------AVP-SKPILK   36
Ashbya gossypii                KSVVFSEEVTS---SPIRATVS-------SSP-VRSAH---------NRPPSKSILK   37
Candida albicans               KSVAFSDDLIS--DIP-------------STPDRNHS------------S-GRSILK   29
Candida dubliniensis           KSVAFSDDLIS--DIP-------------STPERVHS------------P-GRSILK   29
Candida tropicalis             KSVAFSDDLIS--DVP-------------STPEGSQT------------S-GRSILK   29
Debaryomyces hansenii          KSVAFSDDLVS--ELP-------------SSPAAVHCG--SRLPVLEHTP-KKSILK   39
Lodderomyces elongisporus      KSVLFSDDLIL--ELP-------------STPPEKFA-----------TP-KRSILK   30
Scheffersomyces stipitis       KSVAFSDDIAYNDDSP-A---------VVSSPDPRQT------------P-RGSILK   34
Clavispora lusitaniae          KRVAFSDNIAS-DDFP-------------DPFSDNFSD--G------TP-QKSILK   33
Pichia pastoris                KGVKFSEDVQL--SPPKIM----------SSP-RRR--------HTAT----KPILK   32
Pyrenophora teres              KRVDF--KLCD---IPSKSDIAHSWTPTRSSPLRPLPQ------TRLTQP-LKSILK   45
```

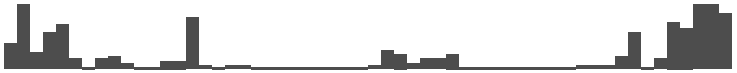

Supplement: Additional file 5 — The N-terminal SILK/PP1 interaction domain of unicellular organisms. The organism name and the length of the domain for each sequence are shown to the left and right of the multiple sequence alignment, respectively. The amino acids are highlighted in different colours based on their property. The degree of conservation at each position in the alignment is represented as bar graph at the bottom of the alignment. [file 1471-2164-13-255-S5.pdf]
