# Supplementary material for: Functional diversification of yeast telomere associated protein, Rif1, in higher eukaryotes
Source: BMC Genomics. 2012 Jun 19;13:255. doi: 10.1186/1471-2164-13-255 (PMC3410773; doi:10.1186/1471-2164-13-255)

Additional file 6

```
                          .    *   .***:               *    : *.*
Aedes aegypti             SPQASP-AASILKRKH-NQDDSG-DDLESPANKRKRVSF   36
Anopheles darlingi        SPQASP-AVGILKRRHGNSDDSD-DENETPANKRKRVSF   37
Anopheles gambiae         SPKASP-AGSILKRRH-NQDESG-DDIDSPVNKRKRVSF   36
Camponotus floridanus     SLTASP-SASILKRQRSSIPEPD-LDSSTP-NKRKRVSF   36
Harpegnathos saltator     SLTASP-SASILKRYRSTVSEQD-SDQITP-NKRKRVSF   36
Nasonia vitripennis       SLTASP-SVSILKR------MQE-VDVETP-NRKKRVSF   30
Drosophila erecta         SPSASP-SVSILKRKLRCESLDD-VTLDSPALKRKRVSF   37
Drosophila melanogaster   SPSASP-SVSILKRKLRCESLDD-VTLDSPALKRKRVSF   37
Drosophila simulans       SPSASP-SVSILKRKLRCESLDD-VTLDSPALKRKRVSF   37
Drosophila ananassae      SPSASP-SVSILKRKLRCESLDD-ATLDSPALKRKRVSF   37
Drosophila sechellia      SPSASP-SVSILKRKLRCESIDD-VTLESPALKRKRVSF   37
Drosophila persimilis     SPSASP-SVSILKRKLRCESIDDSMSYESPALKRKRVSF   38
Drosophila pseudoobscura  SPSASP-SVSILKRKLRCESIDDSMSYESPALKRKRVSF   38
Drosophila virilis        SPSASP-SASILKRKLRCESIDD-F--ESPALKRKRVSF   35
Drosophila willistoni     SPSASP-SASILKRKLRCESIDD-FSMESPAMKRKRVSF   37
Drosophila grimshawi      SPSASP-SASILKRKLRCESMDD-F--ESPAQKRKRVSF   35
Callithrix jacchus        SPLASP-STSILKRGLKR-SQED--EISSPVNKVRRVSF   35
Homo sapiens              SPLASP-STSILKRGLKR-SQED--EISSPVNKVRRVSF   35
Pan troglodytes           SPLASP-STSILKRGLKR-SQED--EISSPVNKVRRVSF   35
Pongo abelii              SPLASP-STSILKRGLKR-SQED--EISSPVNKVRRVSF   35
Macaca mulatta            SPLASP-STSILKRGLKR-SQEE--EISSPVNKVRRVSF   35
Ailuropoda melanoleuca    SPLASP-STSILKRGLKRP-QED--EISSPVHKVRRVSF   35
Equus caballus            SPLASP-STSILKRGLKRPLQED--EISSPVHKVRRVSF   36
Bos taurus                SPLASP-STSILKRGLKRP-QED--EISSPVHKVRRVSF   35
Sus scrofa                SPLASP-STSILKRGLKRP-QED--EISSPVHKVRRVSF   35
Oryctolagus cuniculus     SPLASP-STSILKRGLKRP-QED--ENSSPVNKVRRVSF   35
Mus musculus              SPLASP-STSILKRGLKR-SQED--EI-SPVNKIRRVSF   34
Rattus norvegicus         SPLASP-STSILKRALKR-SQED--EI-SPINKVRRVSF   34
Gallus gallus             SPSASP-STSILKRGVKRH-HED--DSLSPANKIRRVSF   35
Taeniopygia guttata       SPSASP-STSILKRGVKR-SQED--DSLSPANKIRRVSF   35
Xenopus tropicalis        SPSASP-STSILKKGVKRQ-QEN--DSPSPLNKIRRVSF   35
Danio rerio               SPSASP-STSILKKGQKRA-CEE--ETPSPIPKSRRVSF   35
Tribolium castaneum       SPMTVPAS-GILKRK----S-ED--DSGAPVAKRKRVNF   31
Trichoplax adhaerens      SPKTSP--TGILKRW----S---SLDSPSPSGKARRVTF   30
Hydra magnipapillata      SHKMSPA-NGILKKKPN-MA------SP-PITKSRHVTF   30
Ixodes scapularis         DP-ASPGLNGILKRKSCGVDSPE----HSPQRTSRSVSF   34
```

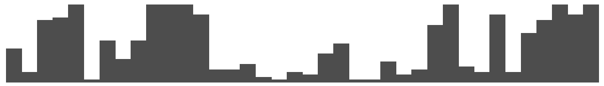

Supplement: Additional file 6 — The C-terminal SILK/PP1 interaction domain of multicellular organisms. The organism name and the length of the domain for each sequence are shown to the left and right of the multiple sequence alignment, respectively. The amino acids are highlighted in different colors based on their property. The degree of conservation at each position in the alignment is represented as bar graph at the bottom of the alignment. [file 1471-2164-13-255-S6.pdf]
