# Supplementary material for: Functional diversification of yeast telomere associated protein, Rif1, in higher eukaryotes
Source: BMC Genomics. 2012 Jun 19;13:255. doi: 10.1186/1471-2164-13-255 (PMC3410773; doi:10.1186/1471-2164-13-255)
Supplement: Additional file 8 — The list of proteins with SILK/PP1 interaction domain inHomo sapiens. The NCBI accession number, protein name, SILK/PP1 interaction domain, protein size, domain length and the position of the motif for the proteins having SILK/PP1 interaction domain are listed in the table. [file 1471-2164-13-255-S8.pdf]

Additional file 8. *Homo sapiens* proteins having SILK/PP1 interaction domain

S.No.	Accession Number	Protein	SILK/PP1 interaction domain	Protein length	Motif length	Position
1	NP_036566.1	sialin	KSVPW(1)PILK	495	10	278-288
2	NP_055178.3	sacsin	KNVRF(3)SILK	4579	12	1989-2001
3	NP_110436.1	cysteine/serine-rich nuclear protein 2	SILK(7)KNVRF	543	16	53-69
4	NP_689974.2	putative uncharacterized protein CXorf58	GILK(9)RRVHF	332	18	11-29
5	NP_000525.1	PMS1 protein homolog 1	GILK(10)KAVIW	932	19	180-199
6	NP_001161693.1	amyotrophic lateral sclerosis 2 chromosomal region candidate gene 11 protein	KNVFF(11)SILK	1820	20	476-496
7	NP_631912.2	sodium/hydrogen exchanger-like domain-containing protein 1	RNVPF(12)SILR	515	21	137-158
8	NP_443173.2	guanylate-binding protein 4	SILR(16)KQVEW	640	25	435-460
9	NP_001008404.1	transmembrane protein C14orf180	SILK(17)RRVWF	160	26	46-72
10	NP_060621.3	telomere-associated protein RIF1	SILK(18)RRVSF	2472	27	2180-2207
11	NP_689822.2	consortin	SILK(19)RRVRF	725	28	621-649
12	NP_001166302.1	zinc finger protein 300	SILK(19)RQVTF	620	28	139-167
13	NP_001073866.1	proton-associated sugar transporter A	GILK(21)RNVTF	748	30	449-479
14	NP_631912.2	sodium/hydrogen exchanger-like domain-containing protein 1	SILR(23)KVVCF	515	32	154-186
15	NP_862830.1	amphoterin-induced protein 2 precursor	KRVVF(26)GILK	522	35	464-499
16	NP_001116848.1	transmembrane protein 72	SILK(28)KQVHF	275	37	188-225
17	NP_733468.2	protein CASC5	SILK(28)RRVSF	2342	37	25-62
18	NP_056175.3	superkiller viralicidic activity 2-like 2	PILK(29)RTVLF	1042	38	461-499
19	NP_008860.4	helicase SKI2W	PILK(29)RTVVF	1246	38	641-679

20	NP_006438.1	ubiquitin carboxyl-terminal hydrolase 16	KAVRF(29)GILK	823	38	288-326
21	NP_059984.2	plexin-A3 precursor	HSVVF(31)PILR	1871	40	425-465
22	NP_001171147.1	ovarian cancer G-protein coupled receptor 1	HRVCF(33)GILR	365	42	169-211
23	NP_596869.4	titin isoform N2-A	RCVAF(34)PILK	33423	43	8526-8569
24	NP_115618.3	plexin-A1 precursor	RTVVF(35)PILR	1896	44	445-489
25	NP_001362.2	dynein heavy chain 8, axonemal	SILR(36)KRVSW	4490	45	4270-4315
26	NP_003553.2	mitochondrial 2-oxoglutarate/malate carrier protein	KSVKF(44)SILK	314	53	21-74
27	NP_068804.1	nuclear receptor subfamily 0 group B member 2	KTVAF(46)SILK	257	55	65-120
28	NP_055875.1	late secretory pathway protein AVL9 homolog	SILK(47)KKVLF	648	56	151-207
29	NP_057396.1	WW domain-binding protein 11	SILK(48)RKVGF	641	57	165-222
30	NP_001121187.1	syntaxin-binding protein 5	HSVAW(48)PILK	1151	57	241-298
31	XP_001717601.2	PREDICTED: hypothetical protein LOC100130701	RNVDF(50)SILK	346	59	174-233
32	NP_001156425.1	synaptotagmin-like protein 2	GILK(55)KHVRF	935	64	272-336
33	NP_079245.2	cysteine/serine-rich nuclear protein 3	GILK(56)KNVHF	585	65	3-68
34	NP_001106203.1	guanine nucleotide exchange factor DBS	GILK(60)KAVLF	1182	69	830-899
35	NP_001116513.2	C-C chemokine receptor type 2	RTVTF(67)GILK	374	76	152-228
36	NP_899243.1	echinoderm microtubule-associated protein-like 5	SILR(69)KHVKF	1977	78	1470-1548
37	NP_000439.1	V(D)J recombination-activating protein 1	KAVRF(70)GILR	1043	79	621-700
38	NP_001093638.1	C-C chemokine receptor type 5	RTVTF(71)GILK	352	80	140-220
39	NP_006717.2	lipopolysaccharide-responsive and beige-like anchor protein	GILR(74)RAVVF	2863	83	1438-1521
40	NP_003316.3	protein HIRA	KVVIW(81)SILR	1017	90	39-129

41	NP_060022.1	ATPase family AAA domain-containing protein 2B	GILR(86)KKVAF	1458	95	373-468
42	NP_995327.1	hypothetical protein LOC389558 precursor	RAVLF(86)GILK	173	95	25-120
43	NP_005500.4	dmX-like protein 1	KAVIW(89)SILR	3027	98	1649-1747
44	NP_115727.3	39S ribosomal protein L45, mitochondrial precursor	KTVRW(91)PILK	306	100	179-279
45	NP_002637.3	phosphatidylinositol-4-phosphate 3-kinase C2 domain-containing subunit beta	GILR(92)RMVIF	1634	101	1017-1118
46	NP_940969.3	hypothetical protein LOC375484	KTVEW(92)GILK	457	101	128-229
47	NP_001380.2	Down syndrome cell adhesion molecule isoform CHD2-42 precursor	PILK(99)RQVAF	2012	108	446-554
48	NP_060931.2	serine/threonine-protein phosphatase 2A 55 kDa regulatory subunit B delta	RVVIF(99)PILK	453	108	57-165
49	NP_001276.2	calcium-activated chloride channel regulator 1 precursor	PILR(100)KQVCF	914	109	620-729
50	NP_001849.2	collagen alpha-1(XIX) chain precursor	PILR(101)KVVEF	1142	110	35-145
51	NP_060814.4	equilibrative nucleoside transporter 3	PILK(101)KTVVF	475	110	300-410
52	NP_056477.1	intraflagellar transport protein 172 homolog	KAVGW(102)PILR	1749	111	1585-1696
53	NP_653267.2	dynein heavy chain domain 1	HCVLW(104)SILR	4753	113	351-464
54	XP_001715812.1	PREDICTED: hypothetical protein LOC390760	RVVLF(104)SILR	387	113	41-154
55	NP_001157101.1	hypothetical protein LOC80133	KKVIF(106)PILK	861	115	328-443
56	NP_001136326.1	hypothetical protein LOC84221	KKVCF(119)GILK	340	128	159-287
57	NP_000312.2	retinoblastoma-associated protein	KNVYF(120)SILK	928	129	289-418
58	NP_001171526.1	poly [ADP-ribose] polymerase 8	KVVIF(127)SILR	854	136	574-710
59	NP_005115.2	nuclear pore complex protein Nup153	SILK(128)RSVYF	1475	137	247-384

60	NP_068798.1	nucleosome assembly protein 1-like 2	PILK(135)RSVLF	460	144	264-408
61	NP_898884.1	sodium/hydrogen exchanger 10	GILK(136)KVVTF	1177	145	747-892
62	NP_056340.2	inhibitor of Bruton tyrosine kinase	PILK(137)KKVSF	1353	146	1084-1230
63	NP_008983.2	NADPH oxidase 1	SILK(137)RKVQF	564	146	412-558
64	NP_057360.2	serine/threonine-protein kinase Sgk2 eta	SILK(139)RAVDW	427	148	130-278
65	NP_570129.1	contactin-associated protein-like 5 precursor	KQVNF(139)GILR	1306	148	279-427
66	NP_055616.3	pecanex-like protein 2	HAVCF(140)PILK	2137	149	1014-1163
67	NP_078954.4	probable asparaginyl-tRNA synthetase, mitochondrial precursor	RSVRF(140)SILR	477	149	9-158
68	NP_150644.1	60S ribosomal protein L8	RAVDF(143)PILK	257	152	30-182
69	NP_004834.1	interleukin-27 receptor subunit alpha precursor	HVVDW(146)PILR	636	155	351-506
70	NP_055983.1	zinc finger CCCH domain-containing protein 4	SILK(152)RTVLW	1303	161	819-980
71	NP_619520.1	putative ATP-dependent RNA helicase DHX30	PILR(156)RNVGF	1194	165	353-518
72	NP_112235.2	mediator of RNA polymerase II transcription subunit 25	RMVQF(159)PILR	747	168	469-637
73	NP_060252.4	hypothetical protein LOC54906	GILR(162)HTVSF	2430	171	1920-2091
74	NP_003857.2	type II inositol-3,4-bisphosphate 4-phosphatase	KKVAF(164)SILR	924	173	690-863
75	NP_004312.2	kinesin-like protein KIF1A	SILR(165)KKVQF	1690	174	576-750
76	NP_898884.1	sodium/hydrogen exchanger 10	KTVTF(170)GILK	1177	179	572-751
77	NP_055704.2	presequence protease, mitochondrial precursor	PILR(173)KAVDW	1037	182	760-942
78	NP_004514.2	kinesin-like protein KIF11	SILK(175)RTVYF	1056	184	646-830
79	NP_002628.2	phosphorylase b kinase regulatory subunit alpha, skeletal muscle	GILR(175)KQVEF	1223	184	831-1015
80	NP_004892.1	ectonucleoside triphosphate diphosphohydrolase 4	KTVSF(176)SILR	616	185	285-470

81	NP_002607.2	period circadian protein homolog 1	RKVAF(178)SILR	1290	187	450-637
82	NP_001006659.1	complement receptor type 2 precursor	PILR(191)HMVRF	1092	200	230-430
83	NP_001868.2	complement receptor type 2 precursor	PILR(191)HMVRF	1033	200	230-430
84	NP_060903.2	lysine-specific demethylase 3A	RQVAW(192)PILK	1321	201	647-848
85	NP_071351.2	family with sequence similarity 38, member B	KAVSF(193)SILK	2752	202	1833-2035
86	NP_003768.2	dynein heavy chain 11, axonemal	SILR(195)KAVKF	4523	204	2247-2451
87	NP_056505.2	serine/threonine-protein kinase 36	KVVDW(197)SILK	1315	206	751-957
88	NP_061841.2	gamma-2-syntrophin	GILR(200)KNVLW	539	209	255-464
89	NP_149075.2	Fanconi anemia group D2 protein	RRVPF(204)SILK	1471	213	986-1199
90	NP_003857.2	type II inositol-3,4-bisphosphate 4-phosphatase	GILK(206)KKVAF	924	215	480-695
91	NP_004949.1	serine/threonine-protein kinase mTOR	PILK(207)KCVQF	2549	216	774-990
92	NP_056146.1	nicastrin precursor	RNVMF(208)SILR	709	217	323-540
93	NP_001786.2	cadherin-5 preproprotein	SILR(210)RNVKF	784	219	296-515
94	NP_001406.1	eukaryotic translation initiation factor 2 subunit 3	HTVRF(212)SILK	472	221	65-286
95	NP_001138295.1	C-myc promoter-binding protein	HKVPF(213)SILK	1906	222	354-576
96	NP_056212.1	DDB1- and CUL4-associated factor 12	PILK(214)RSVSF	453	223	126-349
97	NP_001137148.1	serine/threonine-protein kinase Sgk1	SILK(216)RTVDW	526	225	151-376
98	NP_955352.1	protein Jade-1	GILK(218)RKVNF	842	227	236-463
99	NP_001317.1	cleavage stimulation factor subunit 3	KRVMF(221)SILK	717	230	274-504
100	NP_570720.1	copine-4	KQVQW(224)GILR	557	233	259-492
101	NP_055935.4	Alstrom syndrome protein 1	GILK(225)KVVCF	4169	234	2071-2305
102	NP_003768.2	dynein heavy chain 11, axonemal	KAVKF(230)SILR	4523	239	2446-2685

103	NP_055523.1	kinetochore-associated protein 1	KAVIF(231)SILK	2209	240	1318-1558
104	NP_036221.2	ATP-binding cassette sub-family B member 10, mitochondrial precursor	SILR(237)KNVHF	738	246	254-500
105	NP_061750.1	protocadherin gamma-B7 precursor	RQVLF(241)SILR	929	250	14-264
106	NP_612409.1	nucleolar MIF4G domain-containing protein 1	KNVGF(251)SILK	860	260	507-767
107	NP_055917.1	nephrocystin-4	GILR(252)HMVRW	1426	261	131-392
108	NP_000935.1	serine/threonine-protein phosphatase 2B catalytic subunit alpha	KAVPF(252)SILR	521	261	19-280
109	NP_001157968.1	WD repeat-containing protein 52	PILR(254)RMVNF	1854	263	264-527
110	NP_056365.1	oxysterol-binding protein-related protein 3	GILK(258)HKVYF	887	267	79-346
111	NP_071351.2	family with sequence similarity 38, member B	SILK(264)KCVYF	2752	273	2031-2304
112	NP_789781.2	NACHT, LRR and PYD domains-containing protein 8	KKVMF(265)SILR	1048	274	221-495
113	NP_003244.2	T-lymphoma invasion and metastasis-inducing protein 1	KKVLF(265)SILR	1591	274	1122-1396
114	NP_001007468.1	protein SFI1 homolog	SILR(280)RKVTF	1242	289	667-956
115	NP_001177312.1	bifunctional UDP-N-acetylglucosamine 2-epimerase/N-acetylmannosamine kinase	KHVPF(281)SILR	648	290	280-570
116	NP_056288.1	HEAT repeat-containing protein 5A	RCVSF(293)SILK	1753	302	66-368
117	NP_060647.2	NEDD4-binding protein 2	GILK(298)HTVQF	1770	307	1252-1559

The NCBI accession Number, protein name, SILK/PP1 interaction domain, protein size, domain length and the position of the motif for the proteins having SILK/PP1 interaction domain are listed in the table. The combination of [SPG]IL[KR] followed by [HKR][ACHKMNQRSTV]VX[FW] motif and also the [HKR][ACHKMNQRSTV]VX[FW] followed by [SPG]IL[KR] with the occurrence of up to 300 amino acids in between the motifs were searched in the human protein sequences.
