# Supplementary material for: Functional diversification of yeast telomere associated protein, Rif1, in higher eukaryotes
Source: BMC Genomics. 2012 Jun 19;13:255. doi: 10.1186/1471-2164-13-255 (PMC3410773; doi:10.1186/1471-2164-13-255)
Supplement: Additional file 9 — The list of proteins with SILK/PP1 interaction domain inDrosophila melanogaster. The NCBI accession number, protein name, SILK/PP1 interaction domain, protein size, domain length and the position of the motif for the proteins with SILK/PP1 interaction domain are listed in the table. [file 1471-2164-13-255-S9.pdf]

Additional file 9. *Drosophila melanogaster* proteins having SILK/PP1 interaction domain

S.No.	Accession Number	Protein	SILK/PP1 interaction domain	Protein length	Motif Length	Motif position
1	NP_609685.1	Smg5	SILR(2)KMVDF	1177	11	820-831
2	NP_728174.2	CG32547	PILK(3)KSVYW	1008	12	91-103
3	NP_609597.1	CG9934	SILR(3)RRVQF	1217	12	577-589
4	NP_723391.2	embargoed	KNVDF(11)SILK	1063	20	666-686
5	NP_650002.1	CG6345	RKVHF(16)SILK	583	25	92-117
6	NP_651629.1	huntingtin	PILR(17)KNVIF	3583	26	1870-1896
7	NP_728621.1	CG12090	PILK(18)KKVGF	1544	27	1442-1469
8	NP_725497.1	CG30085	SILK(20)KRVSF	1416	29	1075-1104
9	NP_001097398.1	CG30288	RSVIF(20)SILR	282	29	140-169
10	NP_573095.1	CG9170	GILR(23)KSVRF	1246	32	266-298
11	NP_524465.1	twister	PILK(29)RTVVF	1197	38	591-629
12	NP_524929.1	lethal (2) 35Df	PILK(29)RTVLF	1055	38	477-515
13	NP_001096948.1	CG11203	GILK(31)RSVRF	1272	40	373-413
14	NP_609280.2	CG17005	PILK(39)KMVFW	608	48	40-88
15	NP_733157.1	CG31082	KSVSF(44)GILR	813	53	116-169
16	NP_524613.1	ferrochelatase	PILK(49)RVVLF	384	58	91-149
17	NP_731472.1	slender lobes	KSVDF(49)PILK	1430	58	506-564
18	NP_001137907.1	CG7504	RKVHF(55)SILK	1676	64	913-977
19	NP_610324.2	CG1553	GILK(58)KTVRF	834	67	673-740
20	NP_523965.2	pebble	PILK(59)RSVSF	1311	68	430-498
21	NP_651753.2	CG15523	RSVFW(59)PILR	3731	68	310-378
22	NP_609685.1	Smg5	RKVYF(60)SILR	1177	69	755-824

23	NP_573174.1	CG9104	PILK(71)RMVIF	412	80	270-350
24	NP_649171.2	fat2	SILK(77)KQVNF	4705	86	198-284
25	NP_609706.2	CG15286	RAVEF(80)PILR	511	89	397-486
26	NP_608989.1	Fbw5	SILR(91)RNVFF	656	100	342-442
27	NP_649811.1	CG8032	HQVPF(104)SILR	583	113	134-247
28	NP_001097336.1	NADPH oxidase	SILR(111)RKVDF	1340	120	1091-1211
29	NP_001014689.3	plasma membrane calcium ATPase	GILR(113)KAVMW	1255	122	716-838
30	NP_620474.2	ACXB	PILR(114)KKVCW	1114	123	515-638
31	NP_788719.1	CG7029	SILR(125)KKVSF	1744	134	1384-1518
32	NP_608902.1	CG14040	KQVDF(141)SILK	388	150	155-305
33	NP_609083.2	CG18304	KRVRF(143)SILR	1895	152	628-780
34	NP_648101.1	RhoGEF4	GILR(147)KRVKF	647	156	449-605
35	NP_523704.1	rhomboid-7	PILR(152)KVVCW	351	161	26-187
36	NP_610693.1	CG30035	GILK(153)HAVPF	857	162	208-370
37	NP_477147.2	yin	KSVAF(153)PILR	743	162	34-196
38	NP_788856.1	CG2930	KSVFF(153)PILR	810	162	126-288
39	NP_001097127.1	CG34398	KRVEF(162)GILK	1930	171	710-881
40	NP_524232.2	canoe	SILK(178)KRVSF	2051	187	1720-1907
41	NP_511070.1	sarcoplasmic calcium-binding protein	GILR(208)KAVDF	298	217	9-226
42	NP_726844.1	kin of irre	SILK(216)HKVQF	956	225	251-476
43	NP_730403.2	CG32206	HNVSF(221)PILK	1260	230	808-1038
44	NP_001036490.1	down syndrome cell adhesion molecule, Z	SILR(223)KTVSW	2035	232	149-381
45	NP_477303.1	origin recognition complex subunit 1	SILK(224)KTVSW	924	233	426-659
46	NP_730750.1	CG32462	GILR(226)RNVEF	284	235	25-260

47	NP_611547.2	CG34396	RCVKF(238)SILR	975	247	670-917
48	NP_650953.1	sensory neuron membrane protein	KVVKF(239)SILK	551	248	247-495
49	NP_724537.1	CG30385	KKVNF(250)SILR	536	259	245-504
50	NP_729032.1	guanine nucleotide exchange factor GEF64C	SILR(257)KKVQF	1984	266	318-584
51	NP_651191.1	CG5857	RKVLF(262)SILK	578	271	105-376
52	NP_996345.3	CG42541	RNVSF(273)PILK	1418	282	136-418
53	NP_610172.1	CG30440	SILK(276)KRVLF	1057	285	639-924
54	NP_0010362 87.1	CG34120	SILR(286)RNVNF	1997	295	1377-1672
55	NP_651597.1	CG5508	SILK(287)RQVVF	850	296	203-499
56	NP_650705.1	CG8064	SILR(289)RSVCF	922	298	114-412

The NCBI accession Number, protein name, SILK/PP1 interaction domain, protein size, domain length and the position of the motif for the proteins having SILK/PP1 interaction domain are listed in the table. The combination of [SPG]IL[KR] followed by [HKR][ACHKMNQRSTV]VX[FW] motif and also the [HKR][ACHKMNQRSTV]VX[FW] followed by [SPG]IL[KR] with the occurrence of up to 300 amino acids in between the motifs were searched in the fly protein sequences.
