# Supplementary material for: Functional diversification of yeast telomere associated protein, Rif1, in higher eukaryotes
Source: BMC Genomics. 2012 Jun 19;13:255. doi: 10.1186/1471-2164-13-255 (PMC3410773; doi:10.1186/1471-2164-13-255)
Supplement: Additional file 10 — The list of proteins with SILK/PP1 interaction domain inSaccharomyces cerevisiae. The NCBI accession number, protein name, SILK/PP1 interaction domain, protein size, domain length and the position of the motif for the proteins with SILK/PP1 interaction domain are listed in the table. [file 1471-2164-13-255-S10.pdf]

Additional file 10. ***Saccharomyces cerevisiae*** **protein having SILK/PP1 interaction domain**

S.No.	Accession Number	Protein	SILK motif	Protein length	Motif length	Position
1	NP_010383.1	Hydroperoxide and superoxide-radical responsive glutathione-dependent oxidoreductase; monothiol glutaredoxin subfamily member along with Grx4p and Grx5p; protects cells from oxidative damage; Grx3p	GILR(1)HQVRF	285	10	219-229
2	NP_010188.1	Protein O-mannosyltransferase, transfers mannose residues from dolichyl phosphate-D-mannose to protein serine/threonine residues; acts in a complex with Pmt2p, can instead interact with Pmt3p in some conditions; target for new antifungals; Pmt1p	PILK(2)KVVNF	817	11	616-627
3	NP_012664.1	Cystathionine gamma-synthase, converts cysteine into cystathionine; Str2p	KTVMF(8)SILR	639	17	339-356
4	NP_683715.1	Protein involved in bud-site selection, nutrient signaling, and gene expression controlled by the TOR kinase; diploid mutants display a random budding pattern instead of the wild-type bipolar pattern; Bud27p	PILK(14)KQVGF	796	23	322-345
5	NP_014872.1	Transcriptional repressor involved in regulation of meiosis and silencing; contains WD repeats; Wtm2p	SILK(23)HSVRF	467	32	223-255
6	NP_012485.1	Dead-box family ATP dependent helicase required for mRNA export from the nucleus; co-factor of the exosome complex, required for 3' end formation of 5.8S rRNA;	PILK(29)KTVVF	1073	38	481-519

		Mtr4p				
7	NP_012192.2	Meiosis-specific protein essential for chromosome synapsis, similar to phospholipase A2, involved in completion of nuclear divisions during meiosis; induced early in meiosis; Spo22p	SILR(30)KAVEF	975	39	604-643
8	NP_009545.1	Non-essential transcriptional corepressor involved in the cell cycle-regulated transcription of histone H2A, H2B, H3 and H4 genes; contributes to nucleosome formation, heterochromatic gene silencing, and formation of functional kinetochores; Hir1p	KVVKF(41)SILR	840	50	2-52
9	NP_011422.1	Protein required for accurate chromosome segregation, localizes to the nuclear side of the spindle pole body; forms a complex with Ydr532cp; Spc105p	GILK(49)RRVSF	917	58	21-79
10	NP_010378.1	Aminophospholipid translocase (flippase) that localizes primarily to the plasma membrane; contributes to endocytosis, protein transport and cell polarity; type 4 P-type ATPase; Dnf2p	KNVIF(60)GILR	1612	69	1237-1306
11	NP_009601.1	Meiosis-specific regulatory subunit of the Glc7p protein phosphatase, regulates spore wall formation and septin organization, required for expression of some late meiotic genes and for normal localization of Glc7p; Gip1p	RCVNF(76)SILK	573	85	444-529
12	NP_012954.1	Defining member of the SET3 histone deacetylase	RRVIF(79)PILK	751	88	378-466

		complex which is a meiosis-specific repressor of sporulation genes; necessary for efficient transcription by RNAPII; one of two yeast proteins that contains both SET and PHD domains; Set3p				
13	NP_012036.1	Subunit of the anaphase-promoting complex/cyclosome (APC/C), which is a ubiquitin-protein ligase required for degradation of anaphase inhibitors, including mitotic cyclins, during the metaphase/anaphase transition; Cdc23p	RCVFF(81)SILK	626	90	103-193
14	NP_012160.2	Component of the mitotic exit network; associates with and is required for the activation and Cdc15p-dependent phosphorylation of the Dbf2p kinase; required for cytokinesis and cell separation; component of the CCR4 transcriptional complex; Mob1p	HCVDF(85)PILR	314	94	160-254
15	NP_009385.1	One of two (see also PSK2) PAS domain containing S/T protein kinases; coordinately regulates protein synthesis and carbohydrate metabolism and storage in response to a unknown metabolite that reflects nutritional status; Psk1p	KSVKF(100)SILK	1356	109	662-771
16	NP_010256.1	Dual-specificity kinase required for spindle pole body (SPB) duplication and spindle checkpoint function; substrates include SPB proteins Spc42p, Spc110p, and Spc98p, mitotic exit	KRVSF(101)GILK	764	110	468-578

		network protein Mob1p, and checkpoint protein Mad1p; Mps1p				
17	NP_009834.1	Protein that binds to the Rap1p C-terminus and acts synergistically with Rif2p to help control telomere length and establish telomeric silencing; deletion results in telomere elongation; Rif1p	KSVAF(103)GILR	1916	112	114-226
18	NP_012906.1	Alpha subunit of both the farnesyltransferase and type I geranylgeranyltransferase that catalyze prenylation of proteins containing a CAAX consensus motif; essential protein required for membrane localization of Ras proteins and a-factor; Ram2p	PILK(105)KVVDF	316	114	131-245
19	NP_013534.1	Presumed helicase required for RNA polymerase II transcription termination and processing of RNAs; homolog of Senataxin which causes Ataxia-Oculomotor Apraxia 2 and a dominant form of amyotrophic lateral sclerosis; Sen1p	SILR(125)KHVCF	2231	134	1870-2004
20	NP_010132.1	Transmembrane protein involved in formation of Cvt and autophagic vesicles; cycles between the pre-autophagosomal structure and other cytosolic punctate structures, not found in autophagosomes; Atg9p	SILK(135)RSVIF	997	144	517-661
21	NP_012408.1	Putative histone acetylase, sequence-specific activator of histone genes, binds specifically and highly cooperatively to pairs of UAS elements in core histone promoters,	KNVWF(146)GILK	640	155	100-255

		functions at or near the TATA box; Spt10p				
22	NP_010950.1	Protein of unknown function; authentic, non-tagged protein is detected in highly purified mitochondria in high-throughput studies; GFP-fusion protein is localized to the cytoplasm; transcription induced under conditions of zinc deficiency; Zrg8p	SILR(162)KTVTF	1076	171	800-971
23	NP_011733.1	Voltage-gated high-affinity calcium channel involved in calcium influx in response to some environmental stresses as well as exposure to mating pheromones; interacts and co-localizes with Mid1p, suggesting Cch1p and Mid1p function together; Cch1p	HNVVF(171)SILR	2039	180	369-549
24	NP_012379.1	Probable serine protease of the SPS plasma membrane amino acid sensor system (Ssy1p-Ptr3p-Ssy5p), which senses external amino acid concentration and transmits intracellular signals that regulate expression of amino acid permease genes; Ssy5p	RAVSF(204)PILR	687	213	121-334
25	NP_012030.1	Peroxin required for targeting of peroxisomal matrix proteins containing PTS2; interacts with Pex7p; partially redundant with Pex21p; Pex18p	PILK(211)KHVGF	283	220	43-263
26	NP_010942.1	Gamma subunit of the translation initiation factor eIF2, involved in the identification of the start codon; binds GTP when forming the ternary complex with GTP and	KKVAF(213)SILK	527	222	47-269

		tRNAi-Met; Gcd11p				
27	NP_010954.1	Protein of unknown function, expression is induced by low phosphate levels and by inactivation of Pho85p; Phm8p	RKVFF(215)SILK	321	224	52-276
28	NP_010806.1	Protein disulfide isomerase of the endoplasmic reticulum lumen, function overlaps with that of Pdi1p; may interact with nascent polypeptides in the ER; Eug1p	SILK(218)HHVRF	517	227	77-304
29	NP_012267.1	Member of the DEAH family of helicases, functions in an error-free DNA damage bypass pathway that involves homologous recombination, mutations confer a mutator phenotype; Mph1p	KRVFF(223)PILK	993	232	179-411
30	NP_014759.1	RNA polymerase III subunit C160, part of core enzyme; similar to bacterial beta-prime subunit; Rpo31p	GILK(228)KRVDF	1460	237	139-376
31	NP_014579.1	Mu2-like subunit of the clathrin associated protein complex (AP-2); involved in vesicle transport; Apm4p	SILK(240)RAVKW	491	249	229-478
32	NP_013529.1	Guanine nucleotide exchange factor (GEF) that functions to modulate Rho1p activity as part of the cell integrity signaling pathway; multicopy suppressor of tor2 mutation and ypk1 ypk2 double mutation; potential Cdc28p substrate; Tus1p	KAVRF(250)SILK	1307	259	370-629
33	NP_014864.2	Predicted malonyl-CoA:ACP transferase, putative component of a type-II mitochondrial fatty acid synthase that	SILK(275)RTVQF	360	284	16-300

		produces intermediates for phospholipid remodeling; Mct1p				
34	NP_013496.1	Putative protein of unknown function; green fluorescent protein (GFP)-fusion protein localizes to the cytoplasm; YLR392C is not an essential gene; Ylr392cp	KTVAF(297)PILR	518	306	132-438

The NCBI accession Number, protein name, SILK/PP1 interaction domain, protein size, domain length and the position of the motif for the proteins having SILK/PP1 interaction domain are listed in the table. The combination of [SPG]IL[KR] followed by [HKR][ACHKMNQRSTV]VX[FW] motif and also the [HKR][ACHKMNQRSTV]VX[FW] followed by [SPG]IL[KR] with the occurrence of up to 300 amino acids in between the motifs were searched in the yeast protein sequences.
