# Supplementary material for: Functional diversification of yeast telomere associated protein, Rif1, in higher eukaryotes
Source: BMC Genomics. 2012 Jun 19;13:255. doi: 10.1186/1471-2164-13-255 (PMC3410773; doi:10.1186/1471-2164-13-255)

Additional file 11

```
Callithrix_jacchus                              ALVNCMAPVDIILP---QITSNM-WARGLGQLIRAKN-IKTIGDLSTLTASEIKTLPIRS---PKVSNVKKALRIYHEQQ-----VKSRGLEEI--PVFDISEKTVNGM
Bos_taurus                                      ALVNCVAPVEIILP---QITSNM-WARGLGQLIRAKN-IKTIGDLSTLTASEIKTLPIRS---PKVSNVKKALRIYHEQQ-----VKSRGLEEI--PVFDISEKTINGM
Sus_scrofa                                      ALVNCAAPVDIILP---QITSNM-WARGLGQLIRAKN-IKTIGDLSTLTASEIKTLPIRS---PKVSNVKKALRVYHEQQ-----VKSRGLEEI--PVFDISEKTINGM
Homo_sapiens                                    PLVNCVAPVDIILP---QITSNM-WARGLGQLIRAKN-IKTIGDLSTLTASEIKTLPIRS---PKVSNVKKALRIYHEQQ-----VKTRGLEEI--PVFDISEKTVNGI
Pan_troglodytes                                 PLVNCVAPVDIILP---QITSNM-WARGLGQLIRAKN-IKTIGDLSTLTASEIKTLPIRS---PKVSNVKKALRIYHEQQ-----VKTRGLEEI--PVFDISEKTVNGI
Pongo_abelii                                    ALVNCVAPVDIILP---QITSNM-WARGLGQLIRAKN-IKTIGDLSTLTASEIKTLPIRS---PKVSNVKKALRIYHEQQ-----VKTRGLEEI--PVFDISEKTVNGI
Macaca_mulatta                                  ALVNCVAPVDIILP---QITSNM-WARGLGQLIRAKN-IKTIGDLSTLTASEIKTLPIRS---PKVSNVKKALRIYHEQQ-----VKTRGLEEI--PVFDISEKTINGI
Equus_caballus                                  ALVNCLAPVDIILP---QITSNM-WARGLGQLIRAKN-IKTIGDLSTLTASEIKTLPIRS---PKVSNVKKALRVYHEQQ-----VKSRGLEEI--PVFDISEKTVNGI
Ailuropoda_melanoleuca                          ALVNCVAPVDIILP---QITSNM-WARGLGQLIRAKN-IKTIGDLSTLTASEIKTLPIRS---PKVSNVKKALRVYHEQQ-----VKSRGLEEI--PVFDISEKAVNGI
Oryctolagus_cuniculus                           ALVNCAAPVDIILP---QITSNM-WARGLGQLIRAKN-IKTIGDLSTLTASEIKTLPIRS---PKVSNVKRALRVYHEQQ-----VKSHGLEEI--PVFDISEKTVNGI
Mus_musculus                                    ALVNCAASVDIILP---QITSNM-WARGLGQLIRAKN-IKTIGDLSTLTASEIKTLPIRS---PKVFNVKKALRVYHEQQ-----MKSRGLEEI--PIFDISEKAVNGV
Rattus_norvegicus                               ALVNCAASVDIILP---QITSNM-WARGLGQLIRAKN-IKTIGDLSTLTASEIKTLPIRS---PKVFNVKKALRVYHEQQ-----MKSRGLEEI--PIFDISEKAVNGV
Gallus_gallus                                   ALAGCKAPVDVILP---QITSNI-CARGLGQLIRAKN-IKTVGDLSALTASEIKTLPIRS---PKVSNVKKALRGYHEQQ-----VKSRGCEEI--GALEDGERTVNSA
Taeniopygia_guttata                             ALAGCKAPVDVILP---QITSNI-CARGLGQLIRAKN-IKTVGDLSTLTPLEIKTLPIRS---PKVSNVKRALKGYHEQQ-----VKSRVLEES--TVLEEAEKPGNDV
Xenopus__Silurana__tropicalis                   ALMNCTTPVDVILP---QITSLA-WARGLGQLIRAKN-IKTIGDLSILTPPEIKALPIRS---PKLSTVKKALRVYHEQQ-----TKNKGFDEF--ALLDEIEKPLNVL
Danio_rerio                                     ALVGCSTPVEAVLP---QISSNM-WPRGFGQLVRARN-IKTVGDLSALTPNEIKSLPIRS---PKLSNVRKALKTYHEQQ-----RKGR-SDDL--KSFDEMEKMTSEP
Hydra_magnipapillata                            QLANCSAPIEKILP---SLT----FSRGIGHLVRAQN-ILTIGDLSSLTEMQIENLPIRS---PKVTTVRNALRTYAKGK-----ILPKSPLVI--KVPTENSTSPKED
Trichoplax_adhaerens                            DLVECQEPINQVLP---SLMSSLSCSRGLEQLVRAMN-ICTVGDLCSLSENQVQSLPIRS---PKIPNIKNALRKFQNLQDTKTIVKSNGKIVL--TETDEGNQETDQS
Camponotus_floridanus                           TLSSCAEPIDTIVE---RLTYPL-WKKNLSTHLANRS-LRTIGDLAQLSEREINRLPVKGK--PKTEFVKKVLEHFESTCM----LQAESSDRE--SNAKVQSPIVAMN
Harpegnathos_saltator                           KLSSCREPIDTVID---LLTTPL-WKQNLHTYLTSRN-LLTIGDLAQLSEREINRIPLKGK--PKTEFIRKVLKHYESTSG----SVCRK-ENV--PTLKYMSREKSLD
Nasonia_vitripennis                             SLVSCIDGVETISA---DLVDPL-FTNDLAKHLSVKS-IRSVGDLAQLTERDVNRLPIKS---PKLACIKKALSRYESMKK----LTRTPEKRS--PTPKSSTPLGNKS
Drosophila_sechellia                            SGPRS-DSERLAFV--QQLIVQC--SPGDDLLL------DLIDLLMRTRRERN---------NSS----SKG-VSAVG---------SPDNVAET--AADSSSNL-----
Drosophila_simulans                             SGPRS-DSERLAFV--QQLIVQC--SPGDDLLL------DLIDLLMRTRRERN---------TST----SKG-VSAVG---------SPDNVAET--AADSSSNL-----
Drosophila_melanogaster                         SGPRS-DSERLAFV--QQLMVQC--SPGDDLLL------DLIDLLMRTRRERN---------NSTSTHISKGVVSAVG---------SPDNVAET--AADSSSNL-----
Drosophila_erecta                               SGPRS-DAERLAFV--QQLMVQC--SPDDDLLL------DLIALLMRTRRERKSI-------TNTSTHISKGVVSAVG---------SPDNVAET--AADSSSNL-----
Drosophila_ananassae                            GGPRS-GAERMTFV--QQLLTQC--SPGDDQLL------DLIDLLMRTRRDRNSASKPLPISISVSTSKPGGIGSVIG---------TIDNVAET--AADSSSNL-----
Drosophila_pseudoobscura_pseudoobscura          DILKS-DTERLALV--QQLLLQW--TPSDDQLF------DLINHLIRSRSDRNTN--------------SHSSCSVAVG---------SPDNVAET--AADSSSNL-----
Drosophila_persimilis                           DILKS-DTERLALV--QQLLLQW--TPSDDQLF------DLINHLIRSRSDRNT-----------------SSCSVAVG---------SPDNVAET--AADSSSNL-----
Drosophila_virilis                              AMIRL-ESERLSLL--QQLLQHC--NLGDDQLL------DLIGQLMQLRDN------------------RSNCILASGSAGAG--SINDNVAET--AADSSSNL-----
Drosophila_grimshawi                            AMIRL-EAERFALL--QHLLQQC--SFADDQLL------DLIGQLIRMRDNNPSA--------------ERNNCATAAG-VGNN--VNDDNVAET--AADSSSNL-----
Drosophila_willistoni                           TSSRT-EAERFAFL--QQLLLQC--SFTDEQLL------DIIGQLMVVRDTR-----------------SSNNQNLMG---------HDNVAET--AADSSSNL-----
Coccidioides_posadasii_C735_delta_SOWgp         AVARCFDKCPPDFQ--STLLEKL--QNGLSVWIT-----DAQGQYSATSGSDKCLA------MAVRNIVLASFQSLRSSGS-----TDSQLLNQ--LTPMVASG-----
Coccidioides_immitis_RS                         AVARCFDKCPPDFQ--STLLEKL--QNGLSVWIT-----DAQGQYSATSGSDKCLA------MALTPMIASGFESRHKS-----------IVNS--FIREWNST-----
Aspergillus_oryzae_RIB40                        -MDSHFDSDLPSSP--PELKSQG--ANARNKQISLST-TAVENRVSKKKKAKKSK--------------RSASKDKKRSD-------SQHTQSEE--AREGPSQAG----
Aspergillus_flavus_NRRL3357                     -MDSRFDSDLPSSP--PELKSQG--ANARNKQISLST-TAVENRVSKKKKAKKSK--------------SSASKDKKRSD-------SQHTQSEE--AREGPSQAG----
Aspergillus_clavatus_NRRL_1                     RLSSVKAPSVSAMQ--EAMSTRA--TRSMTSASSQHV-KSSPAPSVAKKRKRQSK--------------TELEQLVDFGS-------QILGSVPA--LEQVTSSN-----
Neosartorya_fischeri_NRRL_181                   RLSSTKTPSAVEVQ--ESMSTRV--SRSMTTASSQNL-KSSPAESVAKRRKRQSK--------------GERGIPAAVIS-------EAL--EPA--KEQGTSQE-----
Pyrenophora_tritici-repentis_Pt-1C-BFP          ALRLLRNNVELCLPSLEAREEDA--DTMVSFYDS-----DTTTEEPKRAFKSPRVK-------ESPFRISKSLR-SKSR-------SPAVPTS---ARRTSSR------
Phaeosphaeria_nodorum_SN15                      ALRRLRNTVELSLPSLPALEGDS--VDGLSFYES-----DSSTEEARPAYKSPRIR-------GSPFKIIKSARKSTTR-------SPALSTPG--SRRTSARQTPKVR
Ajellomyces_dermatitidis_SLH14081               ---SFGSKETPVYP--ENVERAL--RRLKPFVHL-----VLPN-FPPAGDSTIDVP-------SPEF-LSSQEELARSE-------IPINPLEH--TNKICMAF-----
Ajellomyces_capsulatus_G186AR                   ----FGSRTTLEYP--ENVEKAL--RRLKPFVHL-----SLPN-FPQEENSTIDLS-------SPDF-LSSQEDMVESE-------TPIKLLDS--TSKIYKAF-----
Talaromyces_stipitatus_ATCC_10500               QSTEWAQPHVLPQQNIDSTPRKR--LRHDNSQI------QFVTVDPSPRASNMES--------QLLTERQQEVRERQRG-------STSMFLDG--LGSSSPAP-----
Penicillium_marneffei_ATCC_18224                QSTEWGQSHVLPKPKVDSTPRRR--LRHDNSQI------QYVTVDPSPRSSNLES--------QLLTERQHEVKERQRG-------TTSMFLDG--LGSSSPAA-----
Ixodes_scapularis                               ELDACEDPLEKIIP---FMTSSV-WQRSLQRRLSGLN-IRTVRDLAVLTPAQAAQLPIRA---PRVATLRSNLQAYLEAT-----QQAPEADNLG-AGVDDVGADADGP
Magnaporthe_oryzae_70-15                        FFSRCTRQLELQTL------KSF--QPGLVPWIRDED-TRLNSRLSIPVVEIAKSL------WDRVVGLISNMDTHEALP-------LAALEDILCAAFESKHRHIVNS
Tuber_melanosporum_Mel28                        LLKRTQEGVGVWVLDLDHLIKTK-QGEGVVQMLS-AW-TKITDAIVRLPQHDGKIL-----------ADLATLIKCGLES-------RLKPIVNI--SVKSWNLT-----
Aspergillus_niger_CBS_513.88                    EDSAIENELQSSPPKPKSTSPDP--QSNTGNMNTPGS-ANTTSSKNRRSKRRARRS-----------SRQKKAMNAQPR--------QPEPTGES--MTVDSEEPLIKRL
Glomerella_graminicola_M1.001                   VLLAVRPTVDIILP---GLESTR--ETATDENHS--F-VESQDDIEMLTVSSTRS------------SRNRPINFSSPLS-----STKASLSAR--KKLGSTP------
Nectria_haematococca_mpVI_77-13-4               GMEESSGEFGAQAQ---AFVDSQ----EDMSFIALSS-AKSSGHVDQAVSPAPSIT--------KMSLRGASTRKRRRD-------ATPEVVQMKPVKRTNTPR-----
Gibberella_zeae_PH-1                            VVPSTPVDAGSETS---ELPSTT--IRRSKRKRN-----RSDHSVETRSKRRRSTN------------LTGGEKPEDSQ-------PPSQEPNSPIPTVRRSSRRNAGL
Chaetomium_globosum_CBS_148.51                  ---DGKAPKSPVID--CIVVGDS--SQEAEAPLPSRK-TRASSTASAASSSTEPKVR------AGRPKRKRAVSKAQEAG-------PKRPRHDSAEKSEEETASSQTAP
Aspergillus_terreus_NIH2624                     ASLEQPSNLQPPSP---AVDDTPEQISNDGTQIP----LPAVGEPKKRSEVFS--------------MIENLRSSSPAN------TPREFEFM--TPPHLRN------
Clavispora_lusitaniae_ATCC_42720                -FVGQEKSSSDDVQ----LTYES---NSLEHATESNN-TKASTDTTASTSKLKRP-----------IESASTSRRRSKR--------IQTRRAGEI--TELSSDASIVIEI
Lachancea_thermotolerans                        EVAQSKLQASRGAE---ALSEKSTTKGQIKQNVKSQG-VSLNDEVETVAVPSPPKKHESSTTDAAAQIACSKLHENAQKT-----HHGDEVHEKR-SCFEETQPKAASA
Neurospora_crassa_OR74A                         -QASTQEKGKSKLKG--PTSARATAGKGKRKRVASAA-SETAAAAMTPGTGKKRRVG--------RPSSTAPAEQEKSEVQ------VPRSQSQE--VPPKSEPIFYD--
Aspergillus_fumigatus_Af293                     GILAFRRKLLENFQG--SLALWL---RDEARKLN----IESGVESRILTAGDVRTK----------SVSPEGSREAVDMS-------IKSRISYILDDTFEHSRSP----
Sclerotinia_sclerotiorum_1980                   GISGNYRQLPAFIDTQPDCNIPS--EVNLPNLSNKAS-STMIQSHKQKIIGSSPRVVTG---ISKVESRKRSRETTPEPG-------KRKTRKKSSVARLRHDDSQIQFE
Botryotinia_fuckeliana_B05.10                   -SFKGELQYPPMIK---EVLSRL---LPMTDLS---------LASLSESSENQISN------------NHRQPLDIFDTQK------DTNISNEINLPKFSNKAPSSSVI
Pichia_pastoris_GS115                           -TVSLDSLPQSSLP--DTVKISQ--ITSKDLVLTDG---EDEEEITNSTVSPKRKR----------VGNDQQLKKIK----------VDDNFKVH--EIMRNLQRTI---
Vanderwaltozyma_polyspora_DSM_70294             --YSEEKGTPNDLG---LYKEPN--KNSVNVSTGKNS-AKNSIEVNPFEKKSRSP-----------VKVTNSTKGENQTS-----LRANNNEDG--VIISNSEKKTNGN
Candida_glabrata                                SAAHINDHINKNAL---SLEKDK---DNLTNNTEQQ---KNIPNSKSQEISKLSSN----------IADANDLPSNTRES------NCDGSQST--EPAGGKMIKID--
Anopheles_darlingi                              EVKSSRKNLNFSLE----LSKDK--PGESKQSLEKD---QSSEDIIESSQQTTESAR-------SIVGRKVSLRRSRVN-------ESPPMAVT--AVDTVSKSS----
Zygosaccharomyces_rouxii                        RISESPSEKIQEIN-EVQVYATQEKHKVQNEPVKLS--HQTEGQVSLVASEPSSDK-----------KNRPILEEAVIDP-----RSGRGLSSE--DAIDVEDLEPESR
Saccharomyces_cerevisiae_S288c                  DTQSSEVNAYQPIE---VLTSEL--KAVTNRSIKT----NPDHNVVNSDNPLKRPS-----------KETPTSENKRSK-------GHETMVDV--LVSEEQAVSPS--
Tribolium_castaneum                             RVEDSRKNEEVADD---NQKKVP--VITIQKFVQSS--ARTRKNPKPMNKPSFKIK----------LKKRTALVEKKQQT------KTENTESVYDFKADEDTPSDK--
Anopheles_gambiae_str._PEST                     QSKHIFPSEDAVLE---HVMNRY----SLDDMFE-----RYIAAGKTLEKTKTIR------------SLTKELSSKMSDD-----SKTRDIVLD--ELSERHSVEFLD-
Debaryomyces_hansenii_CBS767                    EASSMSQNSINEFD----LVEDE--PSSPPETLD-----RTRKSISPSSNDYSKV-------------IQNGFSSRESSD------SSDDIENT--DDQESTNYKRK--
Kluyveromyces_lactis_NRRL_Y-1140                SIKDSSTEVSECLP---GVANNDEISKNLDTVTS----VNSPSGLPLKIPIFNPFF----------TDVIKLEEDLASK-------GDTYISGK--RSLELMNSAG---
Schizosaccharomyces_pombe_972h-                 LLTSGLKSISKEVV----LAAIKFWNQVFGKFESEEYPIELQKTISYLSKTYIILLPFQSLCPGGKQANHQSSEKMSDILKGVDELRSVSKNGPYASSQDKGEKTTEFS
Sordaria_macrospora                             ALVIIDQDMDMMDP----PSSPPMMEPRRNPLAAEIR--SRSANTSLLDDWQLSSS----------PISGSPLRQQSFAP------VPSSLAVA--AALDAVNAAVGSS
Penicillium_chrysogenum_Wisconsin_54-1255       TIAPSESYTHLVLGNIEAACNGRFSRGSRLELLQQCAELSTTETSAIPCAVQLSEVVWN----ATAQAAADALQSFPMES-------ARGRDGS--VSRDYENVIKI--
Candida_tropicalis_MYA-3404                     VCSLLKDYLAKKMD-----QSDN--YPLIDKLLIGCY-ADLGIDVSNLIGKDCSRFP----------EFRAALEDRGMCL------VEGRIISK--SELESQESTQESI
                                                1.......10........20........30........40........50........60........70........80........90.......100........
```

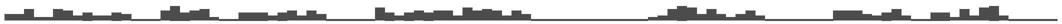

Supplement: Additional file 11 — The DNA binding domain of hRif1 is conserved across the homologues. The organism name and the length of the domain for each sequence are shown to the left and right of the multiple sequence alignment, respectively. The amino acids are highlighted in different colours based on their property. The degree of conservation at each position in the alignment is represented as bar graph at the bottom of the alignment. [file 1471-2164-13-255-S11.pdf]
